# Modelling and testing of a wave energy converter based on dielectric elastomer generators

**DOI:** 10.1098/rspa.2018.0566

**Published:** 2019-02-13

**Authors:** Giacomo Moretti, Gastone Pietro Rosati Papini, Luca Daniele, David Forehand, David Ingram, Rocco Vertechy, Marco Fontana

**Affiliations:** 1 TeCIP Institute, Scuola Superiore Sant'Anna, Pisa, Italy; 2 Institute for Energy Systems, University of Edinburgh, Edinburgh, UK; 3 Department of Industrial Engineering, The University of Bologna, Bologna, Italy; 4 Department of Industrial Engineering, The University of Trento, Trento, Italy

**Keywords:** oscillating water column, dielectric elastomer, wave tank, wave energy converter, dielectric elastomer generator, numerical modelling

## Abstract

This paper introduces the analysis and design of a wave energy converter (WEC) that is equipped with a novel kind of electrostatic power take-off system, known as dielectric elastomer generator (DEG). We propose a modelling approach which relies on the combination of nonlinear potential-flow hydrodynamics and electro-hyperelastic theory. Such a model makes it possible to predict the system response in operational conditions, and thus it is employed to design and evaluate a DEG-based WEC that features an effective dynamic response. The model is validated through the design and test of a small-scale prototype, whose dynamics is tuned with waves at tank-scale using a set of scaling rules for the DEG dimensions introduced here in order to comply with Froude similarity laws. Wave-tank tests are conducted in regular and irregular waves with a functional DEG system that is controlled using a realistic prediction-free strategy. Remarkable average performance in realistically scaled sea states has been recorded during experiments, with peaks of power output of up to 3.8 W, corresponding to hundreds of kilowatts at full-scale. The obtained results demonstrated the concrete possibility of designing DEG-based WEC devices that are conceived for large-scale electrical energy production.

## 1. Introduction

Ocean wave energy is a relevant source of renewable energy that presents attractive attributes such as high potential/concentration and very good predictability [1]. However, the high cost of marine constructions, combined with the difficulty of building a device able to survive in the harsh and aggressive marine environment, has prevented available wave energy converter (WEC) technologies from becoming economically feasible. To date, wave energy conversion technologies are still at the pre-commercial stage, with only a few WECs developed and operated at full-scale [2,3].

Among the numerous WEC architectures, one of the most attractive and extensively investigated is the so-called oscillating water column (OWC) [4]. The interest in such type of WEC is mainly due to its extreme simplicity and minimalist layout. The OWC system consists in a partially submerged hollow structure, with an upper part forming an air chamber and with an immersed part opened to the sea action. The structure partially encloses a column of water which is exposed to the incident wave field at the bottom and to the chamber air pressure at the top. As the waves interact with the OWC structure, wave-induced pressure oscillations at the underwater interface cause the reciprocating motion of the water column, that induces compression and expansion of the air entrapped in the upper chamber. Such oscillating pressure gradient is used to drive a turbo generator which converts the pneumatic power into usable electricity. The OWC operation requires a very limited amount of mechanical moving parts ensuring reliability and reduced/simplified maintenance costs. However, moving parts are required to implement the power take-off (PTO) system, which remains one of the central and critical elements of this type of WECs [5].

Currently, a new class of electro-mechanical transducers called dielectric elastomer generators (DEGs) is being investigated as an alternative PTO technology in WECs [6–12]. DEGs are solid state devices based on soft capacitors which exploit deformation-driven capacitance variations to convert mechanical energy into direct-current electricity [13,14]. DEGs are free from sliding/rolling moving parts and they are made of cheap soft materials that can tolerate harsh ocean environments where steel-made electromagnetic generators struggle. These attributes combined with their high density of converted energy/power per unit mass make DEGs an extremely interesting option to replace conventional PTO technologies in the future.

In earlier works, the combination of DEG–PTO in OWC architectures has been preliminary investigated through theoretical and experimental analysis [11,12], demonstrating the possibility of obtaining promising performance in terms of estimated energy/power output. However, those implementations were based an a very simplified modelling approach and on design solutions which are well conceived for the purpose of small-scale laboratory experiments but are unsuitable to be scaled-up.

**Figure 1.**
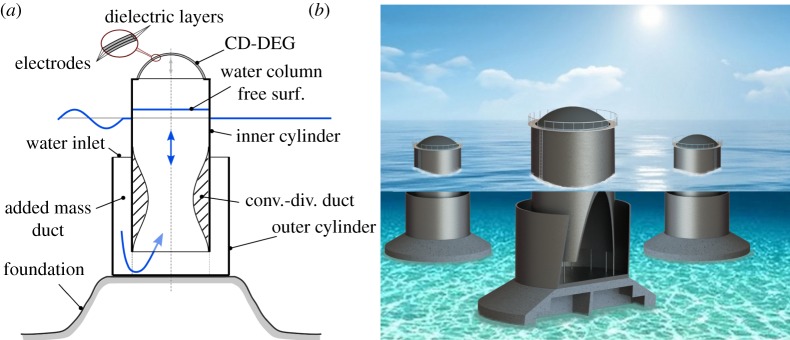
(*a*) Schematic of a bottom-fixed Poly-A-OWC, and detail of the multi-layered DEG architecture. (*b*) Artistic impression of a farm of Poly-A-OWCs and detail of the internal structure of the collector. (Online version in colour.)

In this paper, we introduce a more comprehensive modelling approach and we propose an improved architecture, referred to as polymer-based axial-symmetric OWC, namely Poly-A-OWC, featuring an axial-symmetric U-shaped collector equipped with a circular diaphragm DEG (CD-DEG) [15] at its top (figure 1). In particular, a novel lumped-parameter nonlinear numerical model of the Poly-A-OWC has been developed and employed to run simulations to design/size the geometry of the Poly-A-OWC. Further, an experimental validation of the system has been conducted. A physical prototype of the proposed DEG-based WEC has been built and tested in a wave tank with the aim of practically demonstrating the proposed concept and validating numerical models. The prototype was equipped with a fully-functional CD-DEG PTO. Electrical energy conversion from waves has been successfully demonstrated both in regular and irregular waves, with a remarkable peak power of up to 3.8 W, that corresponds to several hundreds of kilowatts at full-scale, in hydrodynamic similarity conditions.

The paper is organized as follows. Section 2 describes the Poly-A-OWC concept and architecture. Section 3 introduces the integrated multi-physics model of the Poly-A-OWC. Section 4 describes a resonant design of the Poly-A-OWC prototype and provides a set of scaling criteria for the CD-DEG dimensions that enable consistent Froude-scaled dynamics [2]. Section 5 describes the set-up, its control and acquisition electronics, and shows a selection of experimental results, with particular emphasis on electrical power generation tests. Section 7 discusses model validation. Finally, §8 draws the conclusion. Electronic supplementary material is also included which provides details of modelling theory and experimental methodologies.

## 2. The Poly-A-OWC concept

The Poly-A-OWC is an axial-symmetric OWC system that features a U-shaped collector and a CD-DEG fixed on the top of the air chamber as schematically shown in figure 1*a* (an artistic representation is also shown in figure 1*b*). The working principle is similar to that of a conventional OWC but the energy conversion is achieved by exploiting the cyclical deformation of the CD-DEG. Specifically, the wave loads induce pressurization of the air chamber that produce the inflation (or deflation) and the cyclical variation of the capacitance of the CD-DEG. The conversion of the mechanical work done by the air pressure (thus, indirectly, by the waves) to induce the CD-DEG deformation is achieved through an appropriate control of the electrical state of the DEG.

The proposed combination of an axial-symmetric U-shaped collector and a CD-DEG has been conceived with the aim of implementing a DEG-based OWC concept that is dynamically tuned with target wave frequencies, in order to maximize its ability to capture/convert wave energy [16]. In an OWC equipped with a conventional PTO, resonance is typically achieved when the hydrodynamic inertia is large enough to counterbalance the hydrostatic stiffness generated by the gravity forces acting on the water column. In DEG-based OWCs, further hydrodynamic inertia is required to balance the elastic stiffness of the CD-DEG, which adds a contribution to the hydrostatic stiffness.

The collector is based on two coaxial structures: an inner cylindrical shell, which encloses the main water column, and a coaxial outer cylinder. The cylindrical ring volume enclosed between the two cylindrical shells is called an added mass duct, and it has the aim of increasing the inertia of the system. As an additional advantage, this collector geometry has the water inlet section quite close to the free surface, thus providing the water column with large wave excitation forces. The inner cylindrical shell can be equipped with a convergent-divergent (CD) duct (figure 1*a*) to further increase the water flow velocity and increase the hydrodynamic inertia.

Although figure 1 refers to gravity-based bottom-fixed collectors, the Poly-A-OWC may be also employed in a floating moored configuration. Furthermore, even though the PTO in the picture consists of a single CD-DEG, it can also be implemented using several CD-DEGs per device to guarantee smaller size, ease of installation and replacement.

In a number of previous works, similar U-shaped OWC collectors have been investigated in combination with conventional PTO machinery, showing that it is possible to properly size the collector to match the natural frequency of the OWC with a target wave frequency [17–19]. In the following, we show through theoretical and experimental studies that this dynamic tuning can still be achieved in the presence of DEG–PTO systems.

## 3. Mathematical model

Mathematical modelling of coupled DEG–WEC systems represents a challenge, owing to the large-strain electro-mechanical behaviour of DEGs, which results in strongly nonlinear dynamics. A detailed analysis of such a nonlinear response might be addressed through sophisticated computational techniques that employ a local description of the fluid and the elastic continua. However, at design/concept evaluation level, the employment of simpler numerical models providing sufficiently detailed insight into the system behaviour while requiring minimal computational effort is usually preferable.

In this section, an integrated multi-physics model for Poly-A-OWCs is described. The model relies on a set of analytical equations, which allow computationally inexpensive solution of the Poly-A-OWC dynamics, and it is thus especially suitable to perform design and concept evaluation of Poly-A-OWC installations, for which iterative calculations on several design and operating parameters are required. In contrast with other WEC concepts, whose dynamics in working (non-extreme) conditions is well captured by linear dynamical models, Poly-A-OWCs operation is characterized by large deformations of the DEG PTO, which introduce strong nonlinearity in the dynamics. In order to model the essential features of the Poly-A-OWC response, it is thus crucial to isolate and represent the different nonlinear contributions due to the DEG electro-mechanical response and nonlinear hydrodynamic effects in the presence of large water column oscillations. In this regard, an energy balance is used to derive the system's dynamic equations. Such an energy-based approach allows consistent and straightforward integration of the various nonlinear contributions in the model.

The model consists of two blocks: a hydrodynamic sub-model of the U-shaped OWC, and an electro-mechanical sub-model of the CD-DEG PTO. The hydrodynamic sub-model makes use of potential-flow and linear wave theory, and it provides an extension of the traditional modelling framework for WECs, based on Cummin's equation [4,20], by including nonlinear hydrodynamic contributions owing to device geometry and mass transport. An approach similar to that pursued in this paper has been recently proposed in [18], where the authors have derived the OWC equation of motion applying Bernoulli's equation between two points on the fluid volume (one on the OWC inlet section, the other on the water column free surface). Here, in contrast, we obtain the equation of motion from an integral global energy balance on the fluid volume within the OWC collector.

The CD-DEG sub-model relies on the assumption of single degree-of-freedom lumped-parameter kinematics of deformation, and it is built upon a general CD-DEG model introduced in previous papers [12,21]. From a mechanical point of view, the DEG is modelled as a hyperelastic continuum body [13,22], while from an electrical point of view the dielectric elastomer is assumed to be a perfect dielectric, i.e. a perfect insulator free of dielectric losses, and the electrodes resistivity is assumed negligible.

### (a) U-shaped oscillating water column hydrodynamic model

**Figure 2.**
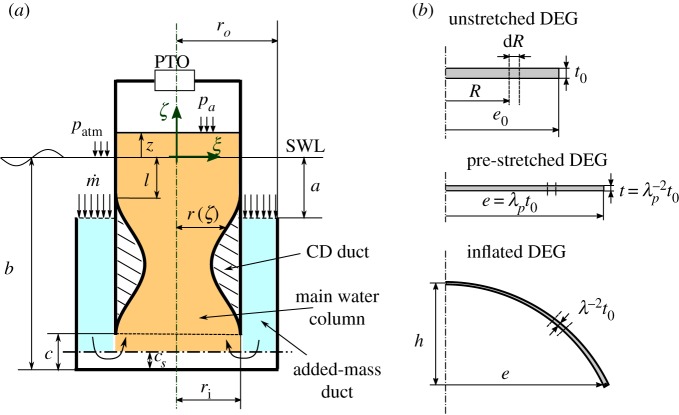
(*a*) Definition of dimensions and control volume of the axial-symmetric OWC collector. (*b*) CD-DEG in the flat unstretched configuration (top), flat pre-stretched configuration (middle), and generic inflated configuration (bottom). (Online version in colour.)

The U-shaped OWC collector geometry and dimensions are schematically represented in figure 2*a*. The collector features:

— An inner tubular thin shell, with radius *r*_*i*_, housing the main water column.
— A horizontal inlet section, located at a depth *a* with respect to the still water level (SWL), through which water enters/leaves the collector.
— An added-mass duct, that drives water from the inlet section to the water column. The duct has outer radius *r*_*o*_ and inner radius *r*_*i*_. The bottom surface of the duct lies at a depth *b* with respect to the SWL.
— An aperture, of height *c*, which connects the added-mass duct to the main water column.
— A CD duct located inside the main water column, having the bottom section in correspondence of the bottom section of the inner cylindrical shell, and the top section at a distance *l* from the SWL.

In accordance with previous studies on OWCs [4,18], we assume that the water column free surface behaves as a rigid piston that remains flat during oscillation. With this assumption, the OWC has one degree of freedom described by the vertical displacement, *z*, of the water column surface from the SWL (*z* is positive for upward displacements).

The water velocity in the various horizontal cross sections of the added mass duct and of the main water column is assumed uniform and perpendicular to the cross-sections. We thus assume that the transversal component of the velocity (lying on the cross-sections plane) is much lower than the axial component, because the slope of the collector walls with respect to the vertical is mild. For simplicity, we also neglect horizontal velocity components in the volume located at the bottom of the collector, in correspondence of the vertical aperture which connects the added mass duct and the water column. Furthermore, we assume that the maximum downward displacement of the water column is smaller (in magnitude) than the distance between the SWL and the CD top section, i.e. *z* > − *l* (a re-formulation of the model without this hypothesis is presented in the electronic supplementary material).

We consider a reference frame *ξ* − *η* − *ζ* with vertical *ζ* axis lying on the device symmetry axis and the origin lying on the SWL. The cross-sectional radius of a generic section of the CD (whose axial coordinate is *ζ*) is indicated with *r*(*ζ*). The water velocity in the main chamber (outside the CD duct) is the same as the water free surface velocity, $\dot{z}$. The velocity in a cross section of the CD duct is indicated with *v*(*ζ*). The uniform velocity in the added mass duct cross sections is indicated by *v*_*i*_. Owing to water incompressibility and mass conservation, the following equalities hold:

3.1 $$\dot{m}=\rho_{w}\pi r_{i}^{2}\dot{z}=\rho_{w}\pi r^{2}(\zeta )v(\zeta )=\rho_{w}\pi \left(r_{o}^{2}-r_{i}^{2}\right)v_{i},$$

where *ρ*_*w*_ is the water density and $\dot{m}$ is the water mass flow rate through the inlet section (positive if entering the collector).

In this analysis, we derive the equations of motion from a global energy balance on the fluid volume within the collector. In accordance with other works on U-shaped OWCs [17,18], we consider a control volume bounded by the OWC lateral walls, the water inlet section, and a horizontal surface located in proximity of the collector bottom (at a generic distance *c*_*s*_ from the collector bottom) below which water velocity can be assumed null, owing to the inversion of the flow direction. For simplicity, in the numerical analyses presented in the paper we use *c*_*s*_ = *c*/2. The influence of this and of other hypotheses on the OWC dynamics representation will be verified *a posteriori* through comparison with experimental results. The control volume is indicated by coloured surfaces in figure 2*a*.

The global energy balance for the control volume reads as follows:

3.2 $${\dot{E}}_{k}+{\dot{E}}_{g}={\dot{W}}_{\mathrm{vh}}+{\dot{W}}_{a}+{\dot{W}}_{\mathrm{in}},$$

where ${\dot{E}}_{k}$ is the time derivative of the kinetic energy, $E_{k}$, of the water within the control volume; ${\dot{E}}_{g}$ is the derivative of the potential gravitational energy, $E_{g}$, of the water within the control volume; ${\dot{W}}_{\mathrm{vh}}$ is the power dissipated by the hydrodynamic viscous forces; ${\dot{W}}_{a}$ is the mechanical power done by air (in the OWC air chamber) on the water column free surface; ${\dot{W}}_{\mathrm{in}}$ is the power associated with the water flow entering the system. The calculation of the different terms is discussed in the following. $E_{k}$

is computed as the sum of the kinetic energy of the fluid in the added-mass duct, that of the fluid in the unrestricted sections of the water column, and that of the portion of fluid within the CD duct:

3.3 $$E_{k}=\pi r_{i}^{2}\rho_{w}\left[\frac{r_{i}^{2}}{r_{o}^{2}-r_{i}^{2}}(b-a-c_{s})+l+z+c-c_{s}+r_{i}^{2}\int_{-b+c}^{-l}\frac{d\zeta }{r^{2}(\zeta )}\right]\frac{{\dot{z}}^{2}}{2}.$$

Choosing the SWL as zero-potential-energy set-point, the potential gravitational energy $E_{g}$ reads as

3.4 $$E_{g}=E_{g,i}+\frac{\pi }{2}r_{i}^{2}\rho_{w}gz^{2},$$

where *g* is the gravity acceleration, $E_{g,i}$ is a constant indicating the potential energy of the control volume portion below the SWL, and the second term accounts for potential energy variations due to the water column surface displacements.

As regards the viscous dissipated power, we define the viscous loss coefficient, *K*_*v*_, relative to the dynamic pressure at the inlet section, thus we obtain:

3.5 $${\dot{W}}_{\mathrm{vh}}=-\dot{m}\frac{K_{v}}{2}v_{i}|v_{i}|=-\frac{\pi \rho_{w}K_{v}r_{i}^{6}}{2{\left(r_{o}^{2}-r_{i}^{2}\right)}^{2}}{\dot{z}}^{2}|\dot{z}|,$$

${\dot{W}}_{a}$ has the following expression:

3.6 $${\dot{W}}_{a}=-\pi r_{i}^{2}p_{a}\dot{z},$$

where *p*_*a*_ is the absolute pressure in the air chamber.

${\dot{W}}_{\mathrm{in}}$ includes the contribution of atmospheric pressure (*p*_atm_), hydrostatic pressure (*ρ*_*w*_*ga*), kinetic and potential volumetric energy density (*e*_*k*_, *e*_*g*_) of the water flowing through the inlet section, and wave-induced pressure (*p*_*w*_):

3.7 $${\dot{W}}_{\mathrm{in}}=\pi r_{i}^{2}\dot{z}\left[p_{\mathrm{atm}}+\rho_{w}ga+\underset{e_{k}}{\underbrace{\frac{\rho_{w}r_{i}^{4}}{{\left(r_{o}^{2}-r_{i}^{2}\right)}^{2}}\frac{{\dot{z}}^{2}}{2}}}\underset{e_{g}}{\underbrace{-\rho_{w}ga}}+\underset{p_{w}}{\underbrace{p_{e}+p_{r}}}\right],$$

where the wave-induced pressure has been expressed as the sum of two contributions, as suggested in [20]: one owing to the wave excitation force (*p*_*e*_), the other owing to radiated waves (*p*_*r*_).

Rearranging equations (3.2)–(3.7), the following dynamic equation is obtained:

3.8 $$M_{z}(z)\ddot{z}=-C_{v}{\dot{z}}^{2}-\pi r_{i}^{2}\rho_{w}gz-\frac{\pi \rho_{w}K_{v}r_{i}^{6}}{2{\left(r_{o}^{2}-r_{i}^{2}\right)}^{2}}|\dot{z}|\dot{z}-\pi r_{i}^{2}p+F_{e}+F_{r},$$

where the following definitions have been introduced:

— *M*_*z*_(*z*) is the generalized inertia of the control volume (reduced to coordinate *z*):
  3.9 $$M_{z}(z)=\pi r_{i}^{2}\rho_{w}\left[\frac{r_{i}^{2}}{r_{o}^{2}-r_{i}^{2}}\left(b-a-c_{s}\right)+l+z+c-c_{s}+r_{i}^{2}\int_{-b+c}^{-l}\frac{d\zeta }{r^{2}(\zeta )}\right].$$
— *C*_*v*_ is a coefficient for the quadratic term owing to variable inertia and mass transportation:
  3.10 $$C_{v}=\frac{\pi }{2}r_{i}^{2}\rho_{w}\left[1-\frac{r_{i}^{4}}{{\left(r_{o}^{2}-r_{i}^{2}\right)}^{2}}\right].$$
— *p* = *p*_*a*_ − *p*_atm_ is the air gauge pressure.
— *F*_*e*_ = *πr*^2^_*i*_*p*_*e*_ and *F*_*r*_ = *πr*^2^_*i*_*p*_*r*_ represent the wave excitation force and the radiation force, respectively.

In practice, it is expected that the OWC will be much smaller than the wavelength, and it will behave as a point absorber. The excitation force can be thus approximated as the sole Froude–Krylov contribution neglecting the diffraction component, as suggested in [23]. Averaging the expression of the pressure due to an undisturbed regular wave (i.e. a sinusoidal wave with height *H* and angular frequency *ω*) [24] over the OWC inlet section, and using the result to approximate *p*_*e*_ leads to the following expression for the excitation force:

3.11 $$F_{e}(\tau )=\pi r_{i}^{2}p_{e}=\pi r_{i}^{2}\frac{\rho_{w}gH}{2}L(\omega )\frac{\cosh(k_{w}(h_{w}-a))}{\cosh(k_{w}h_{w})}\cos(\omega \tau ),$$

where *h*_*w*_ is the water depth at the device location, *k*_*w*_ is the wavenumber (related to the water depth and to the wave frequency through the dispersion relation [24]), time is indicated by *τ* and factor L(ω) comes from an integration on the inlet section and reads as follows (see the explicit calculation in the electronic supplementary material):

3.12 $$L(\omega )=\frac{4}{\pi (r_{o}^{2}-r_{i}^{2})}\int_{0}^{\pi /2}\int_{r_{i}}^{r_{o}}\hat{r}\cos(k_{w}\hat{r}\cos\hat{\theta })d\hat{r}\;d\hat{\theta }.$$

Using equation (3.11), a frequency-dependent excitation coefficient can be defined as

3.13 $$\Gamma (\omega )=\pi r_{i}^{2}\rho_{w}gL(\omega )\frac{\cosh(k_{w}(h_{w}-a))}{\cosh(k_{w}h_{w})}.$$

This coefficient can be used, e.g. to compute the excitation force in the presence of irregular waves made of a superposition of monochromatic waves with heights *H*_*i*_ (generated according to a spectral distribution) and frequency *ω*_*i*_:

3.14 $$F_{e}(\tau )=\sum_{i}\frac{H_{i}}{2}\Gamma (\omega_{i})\cos(\omega_{i}\tau +\varphi_{i}),$$

where *φ*_*i*_ denote the random phases of the different harmonic components.

The computation of the radiated wave load, *F*_*r*_, is non-trivial. It is then convenient, for this contribution, to make use of the linear formulation typically employed in the literature to model radiation. We hereby assume that the radiation force can be written as follows:

3.15 $$F_{r}=-\int_{0}^{\tau }k(\tau -\xi )\dot{z}(\xi )d\xi ,$$

where the convolution kernel *k*(*τ*) has the following expression in the frequency-domain [20]:

3.16 $$K(\omega )=-\omega^{2}\Delta M_{\mathrm{ad}}(\omega )+i\omega B_{r}(\omega ),$$

where Δ*M*_ad_(*ω*) is a frequency-dependent added mass component that asymptotically tends to zero (when *ω* → ∞), and *B*_*r*_(*ω*) is the radiation damping. Usually, the radiation force on a floating body includes a further term beyond the integral term in equation (3.15), which accounts for the hydrodynamic added inertia at infinite frequency [20]. That term is not present here, i.e. we assumed that the asymptotic value of the total system inertia (at large frequency) coincides with *M*_*z*_(*z*) (whose contribution is separately included in equation (3.8)). This assumption is equivalent to neglect the further added mass contribution (at infinite frequency) due to the water outside the collector, and holds for the peculiar application of the U-shaped OWC, where such a contribution is expected to be negligible compared to the large hydrodynamic inertia (*M*_*z*_(*z*)) of the water within the collector. This hypothesis has been found, *a posteriori*, not to significantly compromise the validity of the model.

Owing to the device axial-symmetry, the linearized radiation damping can be computed using Haskind relation [16]:

3.17 $$B_{r}(\omega )=\frac{\omega k_{w}\Gamma^{2}(\omega )}{2\rho_{w}g^{2}\Upsilon },\;\mathrm{with}\;\Upsilon =\left[1+\frac{2k_{w}h_{w}}{\sinh(2k_{w}h_{w})}\right]\tanh(kh_{w}).$$

Similarly, the frequency-dependent component of the added mass can be computed using Kramers–Kronig relation [16]:

3.18 $$\Delta M_{\mathrm{ad}}(\omega )=-\frac{2}{\pi }\int_{0}^{+\infty }\frac{B_{r}(\nu )}{\omega^{2}-\nu^{2}}d\nu .$$

In numerical simulations, the convolution integral in equation (3.15) can be approximated with a linear state-space model to reduce the computational effort [25].

An alternative approach, often used in the literature to model OWCs [4,20], consists in calculating linearized hydrodynamic parameters through a boundary element method (BEM) solver. Compared to the presented model, such an approach does not include nonlinear contributions (which have to be accounted for through appropriate correcting factors) and requires the execution of a BEM software and recomputation of the hydrodynamic parameters every time the OWC collector geometry is updated. By contrast, the BEM approach does not make use of some of the assumptions used in this paper, e.g. negligible horizontal water velocity components, negligible diffraction forces, contribution of the water volume outside the collector to the asymptotic (infinite-frequency) value of the OWC inertia. With the aim of weighting the effect of such assumptions, a comparison between the proposed analytical coefficients and numerical coefficients computed through BEM is reported in the electronic supplementary material.

### (b) Circular diaphragm dielectric elastomer generator electro-hyperelastic model

In order to describe the dynamical response of the CD-DEG, we employ a reduced electro-mechanical model, introduced in [21] and briefly recalled in the following. The model relies on a set of simplifications which provide an analytical description of the DEG pressure-deformation response. The following assumptions are made:

— The deformed CD-DEG has the shape of a spherical cap. It has been demonstrated [21] that using this assumption leads to an accurate description of the DEG in a wide deformation range between the flat equilibrium condition and the hemispheric deformed shape. Thanks to this assumption, it is possible to reduce the CD-DEG continuum model to a single degree-of-freedom model.
— The stretch is equi-biaxial throughout the whole CD-DEG (i.e. the local meridian and circumferential stretches are equal).
— The distribution of the electric field on the CD-DEG is that of a thin parallel-plate ideal capacitor with non-uniform thickness.

A schematic of the CD-DEG assembly is shown in figure 2*b*. The undeformed DEG is a plane membrane (or stack of membranes) with radius *e*_0_ and thickness *t*_0_ (we assume that the entire DEG thickness is made of dielectric material and the electrodes thickness is negligible). Partitioning the DEG thickness into a stack of thin layers (figure 1*a*) allows the implementation of large electric field (which affects the convertible energy density) with limited voltage, and it potentially leads to an improvement in the material dielectric strength [26]. The membrane is uniformly pre-stretched to diameter *e* = *λ*_*p*_*e*_0_ (where *λ*_*p*_ is referred to as the pre-stretch) and clamped to a rigid frame. Elastomeric materials are approximately incompressible, therefore, the thickness of the pre-stretched membrane results in *t* = *t*_0_/*λ*^2^_*p*_. In the inflated configuration, the spherical shape of the membrane is uniquely identified by a single degree of freedom, corresponding to the DEG tip elevation, *h* (positive upwards).

Indicating with *R* the distance of a material point from the symmetry axis measured on the undeformed CD-DEG (figure 2*b* top), the local equi-biaxial stretch of such a point in a deformed configuration (identified by *h*) is as follows [21]:

3.19 $$\lambda (h,R)=ee_{0}\frac{h^{2}+e^{2}}{e^{2}e_{0}^{2}+h^{2}R^{2}}.$$

The volume subtended by the spherical DEG cap in a generic configuration is also a function of *h*:

3.20 $$\Omega_{c}(h)=\frac{\pi }{6}h(h^{2}+3e^{2}).$$

By neglecting the contribution of the DEG mass forces (inertial forces and weight), the following energy balance for the CD-DEG applies:

3.21 $${\dot{E}}_{\mathrm{el}}+{\dot{E}}_{\mathrm{es}}={\dot{W}}_{p}+{\dot{W}}_{\mathrm{es}}+{\dot{W}}_{\mathrm{ve}},$$

where ${\dot{E}}_{\mathrm{el}}$ is the time-derivative of the elastic energy stored in the elastomeric material due to its deformation, ${\dot{E}}_{\mathrm{es}}$ is the derivative of the electrostatic energy stored in the dielectric layers, ${\dot{W}}_{p}$ is the mechanical power of the pressure loads on the DEG, ${\dot{W}}_{\mathrm{es}}$ is the electrical power exchanged by the DEG with the external conditioning circuit (positive if electrical energy is supplied to the DEG), and ${\dot{W}}_{\mathrm{ve}}$ is the power dissipated due to the material viscoelasticity. The calculation of the various terms is detailed in the following.

The CD-DEG is treated as an incompressible hyperelastic continuum body [27], i.e. its local elastic energy density is expressed by a strain-energy function, *Ψ*(*λ*), that depends on the local stretch. The total elastic energy is the integral of *Ψ*(*λ*) over the DEG volume (expressed, e.g. in the undeformed configuration):

3.22 $$E_{\mathrm{el}}=\int_{0}^{e_{0}}2\pi t_{0}R\Psi (\lambda )\;dR.$$

With reference to a hyperelastic Mooney–Rivlin model [27] and to equi-biaxial stretch, *Ψ*(*λ*) has the following expression:

3.23 $$\Psi (\lambda )=C_{1,0}\left(2\lambda^{2}+\lambda^{-4}-3\right)+C_{0,1}\left(2\lambda^{-2}+\lambda^{4}-3\right),$$

where *C*_1,0_ and *C*_0,1_ are constitutive elastic parameters (the small-strain shear modulus of the material is *μ* = 2(*C*_1,0_ + *C*_0,1_)), and *λ* depends on *h* as per equation (3.19).

When a voltage, *V* , is applied on the DEG electrodes, the electrostatic energy stored in the DEG reads as follows:

3.24 $$E_{\mathrm{es}}=\frac{1}{2}CV^{2},$$

where *C* is the CD-DEG capacitance, that can be expressed as a function of the tip height, *h*, as follows [21]:

3.25 $$C\left(h\right)=\frac{\pi \varepsilon n_{L}^{2}\lambda_{p}^{2}e^{2}}{3t_{0}}\left[{\left(\frac{h^{2}+e^{2}}{e^{2}}\right)}^{3}+{\left(\frac{h^{2}+e^{2}}{e^{2}}\right)}^{2}+\left(\frac{h^{2}+e^{2}}{e^{2}}\right)\right],$$

where *ε* is the elastomer dielectric constant (in typical dielectric elastomers, *ε* is two to five times the vacuum permittivity) and *n*_*L*_ is the number of in-parallel dielectric elastomer layers in the CD-DEG assembly.

The mechanical power due to the pressure difference, *p*, between lower and upper DEG faces, reads as follows:

3.26 $${\dot{W}}_{p}=p{\dot{\Omega }}_{c}.$$

Indicating with *Q* the total charge on the DEG in a given configuration, the electrical power supplied to the DEG by the electronics is

3.27 $${\dot{W}}_{\mathrm{es}}=V\dot{Q}=CV\dot{V}+V^{2}\dot{C}.$$

As regards dissipations, visco-elastic models for elastomers have been proposed in [21,28,29] based on rheological representations of the polymeric chains as a combination of elastic and dissipative elements. Such models rely on the definition of additional state variables (namely, the strain rates) and a definition of the material strain-energy function that includes two additive terms for the static elastic response and for viscosity. In the present formulation, the additional term due to viscosity is accounted for by ${\dot{W}}_{\mathrm{ve}}$ in equation (3.21), which can be expressed in accordance with the mentioned formulations [21,28,29]. In practice, full-scale DEG PTOs made of optimized dielectric elastomers are expected to feature low viscosity, and the contributions due to their viscosity are expected to play a minor role. Therefore, for the sake of simplicity and for the scopes of the present formulation, we use a simplified dissipative model, based on the definition of a lumped-parameter material's linear damping, *B*_*h*_, (reduced to coordinate *h*) such that the following equality holds:

3.28 $${\dot{W}}_{\mathrm{ve}}=-(B_{h}\dot{h}){\dot{\Omega }}_{c}.$$

Substituting equations ((3.22)–(3.27)) into equation (3.21) and rearranging the various terms leads to the following equation, that relates the equilibrium pressure, *p*, of the CD-DEG in a certain configuration with tip elevation *h*, voltage *V* and membrane tip velocity $\dot{h}$:

3.29 $$p={\left(\frac{d\Omega_{c}}{dh}\right)}^{-1}\frac{d}{dh}\int_{0}^{e_{0}}2\pi t_{0}R\Psi (\lambda )dR-\frac{V^{2}}{2}{\left(\frac{d\Omega_{c}}{dh}\right)}^{-1}\frac{dC}{dh}+B_{h}\dot{h}.$$

It is worth noticing that the electric field in the dielectric layers is not uniform (as the layers thickness is not uniform). In particular, the electric field, *E*(*R*), in a generic point of the CD-DEG (identified by coordinate *R*) reads as follows:

3.30 $$E(R)=\frac{n_{L}\lambda^{2}(h,R)V}{t_{0}}.$$

Based on equations (3.24) and (3.27), it can be inferred that the instantaneous rate of electrical energy generated by the DEG (either supplied to the power electronics or stored in the DEG electric field) is given by the following expression:

3.31 $${\dot{E}}_{\mathrm{es}}-{\dot{W}}_{\mathrm{es}}=-\frac{V^{2}}{2}\dot{C},$$

which shows that energy is positively generated by the DEG when a voltage is present on the electrodes while the capacitance is decreasing ($\dot{C}<0$).

Coupling between hydrodynamic and CD-DEG model relies on the constitutive model of the closed air volume separating the DEG and the water column. We hereafter assume that air undergoes isentropic transformations. We indicate with *V*_*a*0_ the volume of the air chamber in the equilibrium configuration, when the membrane is flat and the absolute pressure equals the atmospheric pressure, *p*_atm_. In a generic configuration (water column elevation is *z*, DEG tip elevation is *h*), the following equation holds:

3.32 $$p_{\mathrm{atm}}V_{a0}^{\gamma }=(p+p_{\mathrm{atm}}){\left(V_{a0}-\pi r_{i}^{2}z+\Omega_{c}(h)\right)}^{\gamma },$$

where *γ* = 1.4 is the heat capacity ratio of air.

## 4. Design of a small-scale Poly-A-OWC prototype

In order to validate the proposed models and to demonstrate resonant operation of the Poly-A-OWC, a small-scale prototype has been built and tested in a wave tank facility. Owing to the three-dimensional nature of the hydrodynamic problem, the test facility selected for the experiments was the Flowave tank in Edinburgh. Flowave is a circular 2 m-deep basin with a diameter of 25 m, circumferentially ringed by 168 absorbing wave makers [30]. The tank can produce waves with frequency between 0.3 and 1 Hz (peak frequency, if irregular waves), regular wave height up to 0.45 m, or significant wave height up to 0.35 m. Based on the tank operating range, we chose to design a Poly-A-OWC prototype featuring a natural frequency close to 0.5 Hz.

A Poly-A-OWC physical model with a target power of 2÷4 W has been designed and tested at Flowave between May and September 2016. Comparing the wave parameters range used for the tests with typical full-scale waves, a scale factor between 1 : 30 and 1 : 30 can be assumed for the prototype, based on Froude scaling criteria [2]. According to Froude scaling laws, the power of a full-scale system can be estimated multiplying the power of the prototype by the scale factor to the power of 3.5. This provides an equivalent full-scale power output of a few hundreds of kilowatts.

In this section, we describe scaling criteria for the CD-DEG PTO that can be used to project the experimental results to a full-scale scenario, and we report on the design procedure used to guarantee dynamical tuning of the prototype with target incoming waves.

### (a) Consistent circular diaphragm dielectric elastomer generator scaling

In this section, scaling laws for the CD-DEG are established, with the aim of providing a consistently scaled dynamics of the Poly-A-OWC, compliant with the rules of Froude scaling [2]. With the aim of carrying out wave-tank tests on WECs prototypes in conditions of hydrodynamic similarity, Froude scaling provides a set of scaling factors for the different dynamical parameters involved in the tests, which are powers of the WEC's geometric scale factor, *s*_*f*_. Such factors guarantee that the various terms in equation (3.8) scale by the same factor, i.e. *s*^3^_*f*_, with the only exception of the viscous term, which also depends on the Reynolds number.

Proper scaling of the CD-DEG PTO relies on the observation that the relative air pressure in the chamber, *p*, must scale proportionally with *s*_*f*_.

Based on the CD-DEG equilibrium equation (namely, equation (3.29)), indicating with *s*_*f*_ the geometric scaling factor for the WEC dimensions, we assume the following set of scaling criteria for the DEG:

— The employed elastomeric material is the same at different scales.
— The DEG radial sizes (*e* and *e*_0_) scale with *s*_*f*_. In this way, if the CD-DEG displacements (e.g. *h*) scale with *s*_*f*_, the stretch and the strain-energy density (see equations (3.19) and (3.23)) are scale-invariant. The subtended cap's volume (*Ω*_*c*_) scales with *s*^3^_*f*_.
— The applied voltage is scaled in a way that the electric field, *E*, in the CD-DEG (equation (3.30)) is the same at different scales. As a consequence, the electrostatic contribution in equation (3.29) results independent on the number of dielectric layers, and different values for *n*_*L*_ can be used at different scales.
— Owing to the mentioned assumptions and in order for the equilibrium pressure, *p*, to scale with *s*_*f*_, it is necessary that the CD-DEG thickness, *t*_0_, scales with *s*^2^_*f*_, rather than with the geometric scale factor, *s*_*f*_. This choice indeed guarantees the elastic and electrostatic terms in equation (3.29) to scale consistently.
— The dissipative term in equation (3.29) does not scale consistently. In effect, wave frequencies at tank scale are larger than at real scale, thus resulting in larger strain rates, with consequent greater influence of visco-elastic dissipations. Despite this inconsistency, the effect of visco-elastic losses is expected to be almost negligible in optimized materials for PTO application.

In order to achieve a consistent scaling of the device dynamics, the air chamber compressibility should also be properly scaled [31]. In small-scale devices, this can be achieved by extending the prototype air chamber with an external reservoir, thus adding technical complexity. As the main focus of the present paper is to explore the performance of a small prototype and validate models, air chamber compressibility scaling has not been implemented in order to limit the prototype complexity.

### (b) Dynamically tuned design

Based on linear formulations, a WEC achieves maximum power output in the presence of a given sea state in conditions of resonance with the incident waves [16]. For a nonlinear system, the identification of optimality conditions for power output maximization is a complicated task which requires appropriate optimization methods. However, nonlinear WEC systems are likely to show resonant-like behaviour over certain frequency ranges, and, by analogy with the linear case solution, their power conversion capability is expected to be enhanced in such conditions. Based on this observation, a design procedure is here described featuring a resonant-like behaviour in the testing frequency range.

**Figure 3.**
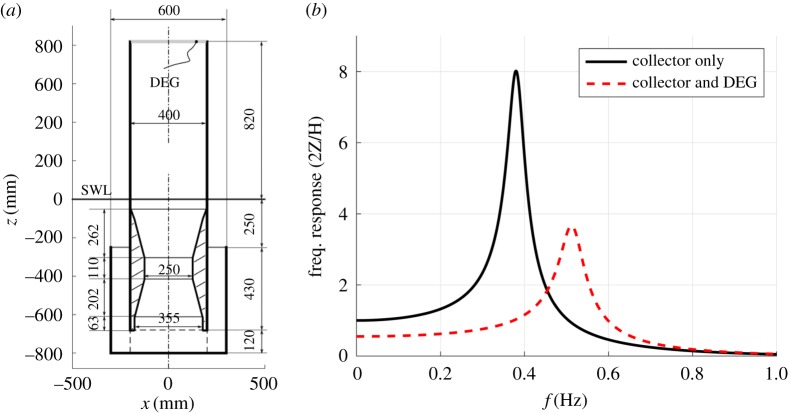
(*a*) Geometry and dimensions of the axial-symmetric OWC prototype collector. (*b*) Mechanical frequency response of the OWC collector (open to atmosphere) and of the overall Poly-A-OWC system. (Online version in colour.)

With the aim of designing a prototype with a target natural frequency of approximately 0.5 Hz (i.e. centred within the operating frequency range of the tank), we considered the purely mechanical response of the CD-DEG, in the absence of control and electrical activation. With the aim of representing the device frequency response, we linearized the presented set of equations, i.e. we neglected quadratic terms in equation (3.8) and calculated position-dependent coefficients at *z* = 0. As for the DEG, we neglected DEG viscosity and linearized the resulting algebraic relation between free surface position, *z*, and pressure, *p*. Based on such a linearized model, which allows time-inexpensive iteration on the design parameters, we selected the prototypes dimensions shown in figure 3*a* for the collector, including a piecewise linear CD duct, and the features shown in table 1 for the CD-DEG. In practice, DEGs with slightly different features (e.g. thickness) have also been employed in the experiments. The choice of the collector dimensions results from a compromise between target dynamical behaviour of the prototype and availability of commercial components (e.g. pipes and reductions) to build the setup. For this reason, e.g. the CD duct has the top section relatively close to the SWL, so that, in certain operating conditions, the water column free surface can intersect the CD cross sections.

**Table 1.** Parameters of the nominal CD-DEG PTO prototype. The reported hyperelastic parameters might differ from those found in other papers since they have been obtained by fitting experimental data acquired on specimens tested with different states, ranges and rates of the imposed deformation and subjected to different pre-conditioning cycles to remove stress-softening effects.

material	acrylic VHB 4905
hyperelastic parameters	*C*_1,0_ = 5500 Pa, *C*_0,1_ = 570 Pa (*μ* = 12.1 kPa)
dielectric constant	*ε* = 4.2 · 8.85 · 10^−12^ F m^−1^
DEG stretched radius, *e*	195 mm
DEG unstretched thickness, *t*_0_	2 mm
pre-stretch, *λ*_*p*_	3.5
thickness in the flat configuration, *t* = *t*_0_/*λ*^2^_*p*_	0.16 mm
No. of in-parallel dielectric layers	2

The nominal CD-DEG is made of a commercial acrylic elastomer, namely VHB 4905 (by 3M), whose electro-mechanical properties are listed in table 1. A wider description of this material's physical parameters, including a viscosity characterization, is provided in [10,21]. VHB is widely employed as dielectric elastomer in small-scale experiments as it is easy to handle, pre-stretch and stack [32,33]. In practice, since this material is not specifically conceived for dielectric elastomer application (it is an industrial tape), it presents a number of drawbacks, such as large visco-elasticity and electrical dissipation, that make it unattractive for real-scale applications. According to the scaling rules established in §4a, assuming a prototype scale factor of 1/30–1/20, the DEG dimensions in table 1 correspond to a full-scale CD-DEG radius of 3.9–5.85 m and a thickness of the pre-stretched stack (in the flat configuration) of 7–15 cm.

Based on available assessments of the energy density that the DEG can convert in a cycle (in [33], e.g. energy densities up to 0.1 J g^−1^ were measured on a DEG with a similar topology made of the same acrylic material), it is expected that power outputs consistent with the target of a few Watts can be produced at the considered wave frequencies.

Owing to the non-optimal employed dielectric elastomer material and to the lack of a consistent scaling of the air stiffness (see §4a), the prototype should not be looked at as the exact small-scale equivalent of a hypothetical full-scale device, but rather as a demonstrative implementation of a scaled Poly-A-OWC.

As a result of the design procedure, figure 3*b* shows the amplitude of the water column oscillations (per unit of wave amplitude) as a function of the frequency, for the open OWC collector (with no CD-DEG on top) and for the complete Poly-A-OWC (collector closed at its top by the DEG).

The frequency response in the two configurations clearly shows that the CD-DEG is responsible for an increase in the OWC natural frequency and a decrease in the water column oscillation amplitude, due to its rigidity.

Although relevant nonlinearities are expected to be present in practice, which would lead, e.g. to a reduction of the maximum oscillation amplitudes, figure 3*b* provides a sufficient confidence on the Poly-A-OWC prototype resonant operation at the target frequency close to 0.5 Hz.

## 5. Experimental tests

This section describes construction and testing of the Poly-A-OWC prototype and its electronics conditioning circuit, and measurement procedures for the relevant variables (air chamber pressure, water column elevation, CD-DEG deformation, electrical variables).

**Figure 4.**
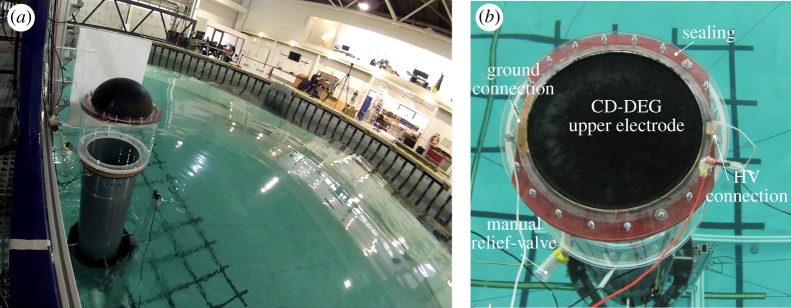
(*a*) Shot of the Poly-A-OWC prototype during wave tank tests. (*b*) Top view of a CD-DEG sample mounted on top of the Poly-A-OWC prototype. (Online version in colour.)

The Poly-A-OWC prototype has been manufactured using commercial PVC pipes for the collector coaxial shells and CD duct, and custom-made acrylic parts for the connection flanges and for the upper part of the air chamber. A picture of the device in operation in the tank is shown in figure 4*a*.

Several CD-DEG samples have been built by pre-stretching the dielectric layers on rigid acrylic frames and coating them with electrodes. Each sample includes two stacked dielectric elastomer layers and three electrodes: two on the stack outer faces, and one in between the two layers. Outer electrodes are grounded, while high voltage (HV) is applied on the central electrode, which is insulated from the environment for safety and to prevent charge dispersion in air. With reference to the CD-DEG nominal design (table 1), each layer has unstretched thickness of 1 mm, and is obtained by bonding two VHB 4905 layers on top of each other. In the experiments, thicker layers have also been tested, up to a total thickness *t*_0_ = 3 mm (1.5 mm per layer).

Electrodes are made of conductive grease (MG-Chemicals 846), and they are connected to the wires of the circuit by means of a copper foil connecting the electrodes perimeter to the circuit terminals. Although conductive grease is unsuitable for the final full-scale application, it is widely used in experiments [32,34] to simplify the manufacturing. A picture of a CD-DEG assembly equipped with electrical connections and mounted on the OWC collector is shown in figure 4*b*.

### (a) Electronics and control

**Figure 5.**
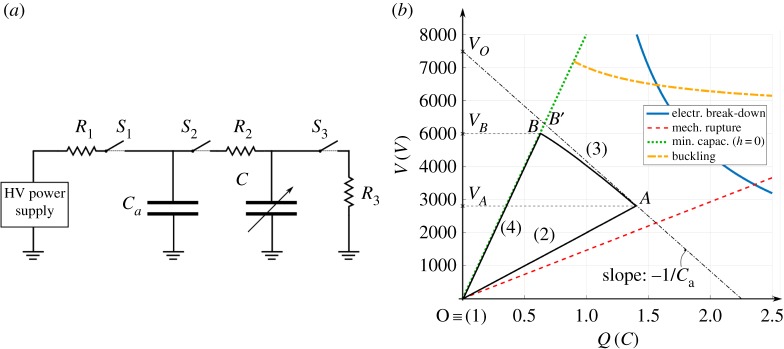
(*a*) Conditioning circuit for the DEG PTO. (*b*) Charge-voltage, *Q* − *V* , plane representing the CD-DEG operating constraints (namely, electrical break-down, mechanical rupture, minimum capacitance configuration, electro-mechanical buckling) and example of conversion cycle (loop OABO). (Online version in colour.)

The conditioning circuit layout is shown in figure 5*a*, and it includes: three resistors (*R*_1_, *R*_2_ and *R*_3_), three HV switches, *S*_1_, *S*_2_ and *S*_3_ (HM12-1A69-150 by MEDER electronic), and two capacitors: the CD-DEG (with variable capacitance, *C*) and an in-parallel capacitor with constant capacitance *C*_*a*_. A HV power supply (10/10B-HS by TREK) is used to activate the DEG at each cycle.

Control of the CD-DEG is performed based on a cyclic sequence of operations, similarly to what is described in [21]. The CD-DEG is kept electrically active only while its capacitance is decreasing (as suggested by equation (3.31)), and the sequence of operations composing the control cycle is as follows:

(1) *Expansion phase*. At the beginning of each cycle (while the DEG is expanding upward or downward), *C*_*a*_ is charged to a fixed voltage, *V*_0_, by the power supply (during this phase, *S*_1_ is kept closed).
(2) *Priming phase*. When the DEG capacitance reaches a maximum, *S*_1_ is opened, *S*_2_ is closed, and *C*_*a*_ reaches an equilibrium voltage, *V*_*A*_, with the DEG (almost instantaneously).
(3) *Harvesting phase*. As the DEG moves towards the flat configuration (i.e. its capacitance decreases), *S*_2_ remains closed and the parallel *C*_*a*_ and *C* is isolated from the external supply (i.e. the total charge is constant, except for the charge losses through the dielectric layers).
(4) *Discharging phase*. When the CD-DEG is flat, *S*_2_ is opened, *S*_3_ is closed and the DEG is fully discharged on resistor *R*_3_. Switch *S*_3_ is then opened and the successive cycle is started. The DEG capacitance in the flat configuration is indicated by *C*_*B*_, and *V*_*B*_ is the corresponding voltage on the CD-DEG and *C*_*a*_.

The use of the in-parallel capacitance, *C*_*a*_, has two motivations. On the one hand, priming the CD-DEG using the in-parallel capacitor rather than directly with the power supply allows an accurate estimate of *C*_*A*_ (the DEG capacitance at priming), which can be obtained from the measured equilibrium voltage between the DEG and *C*_*a*_ after priming:

5.1 $$C_{A}=C_{a}\left(\frac{V_{0}}{V_{A}}-1\right).$$

Furthermore, increasing the overall capacitance of the parallel *C*-*C*_*a*_, allows the conversion of large amounts of electrical energy while limiting the voltage rise on the CD-DEG, as suggested in [34].

The control cycle can be conveniently represented on a diagram holding on the axes the charge, *Q*, and voltage, *V* , on the DEG, as shown in figure 5*b*. The graph shows a set of curves representing the operating constraints of the reference CD-DEG (as described in [35]), and a closed loop (solid black line) made of a set of curves representing the mentioned four-phase control cycle. In particular, expansion phase (1) corresponds to the purely mechanical DEG expansion, and is trivially represented by a point on the axes origin. Priming phase (2) is represented by line OA, which is an iso-capacitance curve (*C* = *C*_*A*_). Harvesting phase (3), during which the DEG capacitance decreases and energy is transferred from the DEG to *C*_*a*_, is ideally represented by a straight line (AB' in the plot) with the following equation:

5.2 $$C_{a}V+Q=C_{a}V_{0},$$

that expresses charge conservation on the capacitors parallel. In practice, due to leakage currents through the dielectric layers, this phase is represented by curve AB. Finally, discharging phase (4) corresponds to iso-capacitance line BO (*C* = *C*_*B*_).

The different phases (1)–(4) are triggered based on the air chamber gauge pressure measurements: when the CD-DEG is maximally inflated upward/downward, the pressure is maximum/minimum. When the CD-DEG is flat, the gauge pressure is zero. The energy harvesting controller is implemented on a real-time machine (Performance real-time target machine by SpeedGoat), running at a sample frequency of 10 kHz, via the Matlab Real-Time software environment. Resistances *R*_1_, *R*_2_ and *R*_3_ are introduced to prevent current peaks. Each of their values (*R*_1_ = *R*_3_ = 100 kω, *R*_2_ = 50 kω) is chosen as the minimum that guarantees safe operation of the switches while introducing a negligible effect on the estimate of the generated energy. To prevent DEG activation in the cycles where the membrane deformation are very small (especially in irregular waves), and generated energy would not compensate the losses, a threshold on peak pressure is set: when the pressure time-series reaches a maximum/minimum but its magnitude is below 150 Pa, the CD-DEG is not activated.

### (b) Testing procedures and data acquisition

Different types of experiments were carried out, including purely hydrodynamic tests with air chamber at atmospheric pressure (with no DEG, and collector open towards atmosphere), and electro-mechanical tests on the Poly-A-OWC with DEG. Each test had a first phase in which the system was tested mechanically (without electric activation), and a second phase with actual electrical control.

Tests have been carried out both in regular (monochromatic) and irregular (panchromatic) waves with JONSWAP spectral distribution [2]. The following wave parameters have been employed: wave frequencies (peak frequencies, if irregular waves) between 0.3 and 0.7 Hz (i.e. 0.05–0.15 Hz full-scale equivalent, assuming a scale factor of 1/30–1/20); wave heights (significant wave height if irregular waves) of 0.1–0.25 m (2–7.5 m at full-scale).

The following parameters have been varied throughout the different test runs: (1) wave parameters (wave frequency and height, spectral parameters); (2) CD-DEG thickness *t*_0_; (3) in-parallel capacitance *C*_*a*_ and (4) level of the electrical load, expressed by the charging voltage *V*_0_. The resulting maximum electric field on the DEG prototypes throughout the different tests was in the order of 150 MV m^−1^. The obtained results thus provide a measure of the maximum performance achievable with the considered dielectric material compatibly with its break-down limits [10].

Relevant variables were measured as follows. The undisturbed wave elevation in the device far-field and the water column elevation were measured with resistive wave-gauges by Edinburgh Designs using a custom driver (by Edinburgh Designs) and a National Instrument acquisition system available at Flowave. The air chamber gauge pressure was measured with a piezo-resistive sensor (MPX12 by Freescale Semiconductor). Voltages on CD-DEG, in-parallel capacitor and power supply output were measured with custom made high-impedance (10 Gω) probes driven by the same electronics used to control the CD-DEG. The membrane deformed shape was monitored through a high-speed camera (Point Grey GS3-U3-23S6M-C with lens 250F6C, using acquisition software FlyCapture 2.9). Image post-processing has been carried out using computer-vision techniques, that provided a set of time-series for the membrane tip elevation, *h*(*τ*) (see the details in the electronic supplementary material).

The different measured signals were synchronized using analog trigger signals.

## 6. Experimental results

This section reports on experimental results and performance assessment of the reference Poly-A-OWC prototype. First, some relevant time-series describing the device dynamics in different scenarios are reported. With reference to electricity generation experiments, a procedure to estimate the electrical power generated by the device is described, and estimates of the electrical power outputs generated in different experimental sets are reported.

### (a) Relevant time-series and dynamical response

In the following, we present relevant time-series aimed at comparing the prototype response in different scenarios.

**Figure 6.**
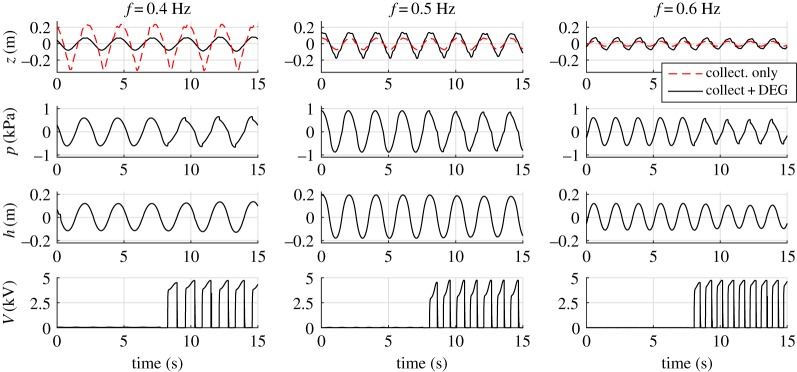
Experimental time-series of water column displacement (*z*), air chamber gauge pressure (*p*), DEG tip elevation (*h*) and CD-DEG voltage (*V* ) for three tests featuring same wave height (*H* = 0.15 m) and different wave frequencies. The water column displacement is shown for both the cases with and without the DEG (water column open to the atmosphere). (Online version in colour.)

With reference to regular waves featuring the same height (*H* = 0.15 m) and different wave periods, figure 6 shows relevant time-series for the case with atmospheric air chamber (no membrane), and with a DEG sample installed. In the first case, the only relevant variable is the water column elevation (*z*) inside the collector (red dashed line). In the second case, the time-series of air gauge pressure, *p*, membrane tip displacement, *h* and CD-DEG voltage, *V* , are also shown. The DEG was electrically activated only during the second half of the considered time lapse. In this example, the DEG unstretched thickness was *t*_0_ = 2 mm. With reference to the electrically active phase, the in-parallel capacitance was *C*_*a*_ = 394 nF and the charging voltage was *V*_0_ = 6 kV.

In the two cases (with and without the CD-DEG), the two time-series of *z* are shifted with respect to each other in order to align their maxima/minima.

A comparison of the plots shows that:

— In the presence of the DEG membrane, the oscillations have maximum amplitude at the intermediate frequency of *f* = 0.5 Hz, that is, indeed, the design resonance frequency;
— Free surface oscillations in the absence of the membrane are largest at the lowest frequency, in agreement with the design forecast in figure 3*b*. This confirms that the CD-DEG is responsible for a shift of the resonance peak towards larger frequencies.
— As expected, when the OWC resonates in the absence of the DEG membrane (around *f* = 0.4 Hz), the free surface oscillations are larger than those of the coupled resonating collector+DEG system (at 0.5 Hz).
— Quick electric activation provokes a jump in the air pressure. This is due to the electric-field-induced membrane expansion, that, despite small (no jump in *h* is indeed visible), results in a significant air pressure drop due to the high air rigidity at the experiment scale.
— The oscillation amplitudes of *z*, *p* and *h* change before and after the activation phase. In particular, the oscillation amplitude in the presence of activation decreases for *f* = 0.5 and 0.6 Hz, while it does not visibly vary for *f* = 0.4 Hz. The electric activation has two effects on the WEC dynamics: (1) it damps the WEC oscillation, as it subtracts mechanical energy from the system; (2) it makes the DEG rigidity decrease, as it induces a reduction in the material stress [13], thus reducing the system natural frequency. When the DEG is activated, the oscillation amplitude at wave frequencies *f*≥0.5 Hz decreases, both because the system is damped and the natural frequency becomes further lower than the excitation frequency. At 0.4 Hz, owing to the electric activation, the natural frequency gets closer to the excitation frequency, thus compensating the increase in damping.

**Figure 7.**
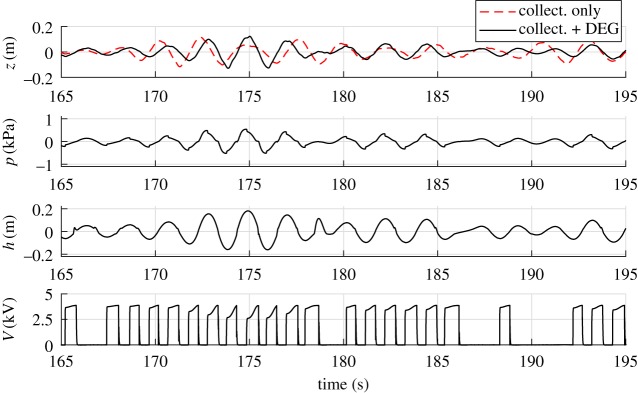
Experimental time-series of water column displacement (*z*), air chamber gauge pressure (*p*), membrane tip elevation (*h*) and DEG voltage (*V* ) for an irregular sea state featuring significant wave height *H*_*s*_ = 0.15 m, and peak frequency *f*_*p*_ = 0.5 Hz. Red dashed lines refer to the case with atmospheric air chamber (no CD-DEG) and black solid lines are for the case with CD-DEG. (Online version in colour.)

With reference to an irregular wave test (irregular waves with JONSWAP spectrum with peak-enhancement factor *γ*_*s*_ = 3.3, significant wave height *H*_*s*_ = 0.15 m and peak frequency *f*_*p*_ = 0.5 Hz) the time-series of *z*, *p*, *h* and *V* relative to the cases without the CD-DEG (atmospheric air chamber) and with the CD-DEG are shown in figure 7. The phases of the two signals are such that the incoming waves (measured by a far-field wave gauge) are the same. The CD-DEG parameters and *C*_*a*_ are the same as in the previous example. The charging voltage (when electric activation is present) is *V*_0_ = 5 kV.

In the presence of the CD-DEG, the water column elevation profile is significantly different than for the atmospheric air chamber, due to the relevant modification in the frequency response introduced by the CD-DEG. As in the regular wave case, the effect of quick electric activation is clearly visible from the pressure time-series, as it results in a pressure drop. It is worth noticing that the control does not activate the membrane during certain cycles, when the pressure magnitude is below the threshold value of 150 Pa.

### (b) Power conversion assessment

With reference to power generation tests, in which the electrical state of the DEG is actively controlled, we hereby present a procedure for the estimate of the electrical energy converted by the system and an evaluation of the electrical power output performance of the prototype in different operating conditions.

The electrical energy generated by the CD-DEG in a cycle, $W_{u}$, can be estimated as the difference between the energy harvested from the DEG and *C*_*a*_ during the discharging phase (phase (3) in figure 5*b*) and that spent to charge the system (phase (1)):

6.1 $$W_{u}=\frac{1}{2}C_{B}V_{B}^{2}-\frac{1}{2}C_{A}V_{A}^{2}+\frac{1}{2}C_{a}(V_{B}^{2}-V_{A}^{2}),$$

where *V*_*A*_ and *V*_*B*_ are the voltages (on the DEG and *C*_*a*_) immediately after electric priming and before discharging respectively (figure 5*b*), measured by the HV probes. Capacitance *C*_*A*_ depends on the DEG deformation at the current cycle and is estimated using equation (5.1) and capacitance *C*_*B*_ at the discharging instant is known from direct measurement on the flat DEG stack.

The first term in equation (6.1) is the energy recovered from the DEG during discharging phase (4) (figure 5*b*). The second (negative) term is the electrical energy supplied to the CD-DEG during priming (2). The third term is the energy transferred from the DEG to *C*_*a*_ during harvesting phase (3).

In this estimate, it has been assumed that the DEG capacitance remains constant during the charging phase (which is almost instantaneous), i.e. voltage-induced expansion of the CD-DEG during priming is considered negligible (see the electronic supplementary material for discussion). It is important to point out that the employed electric set-up (figure 5*a*) is conceived to provide an accurate estimate of the DEG generated energy. However, the circuit does not allow the delivery and storage of such generated energy (e.g. the generated power is dissipated through the resistors).

**Figure 8.**
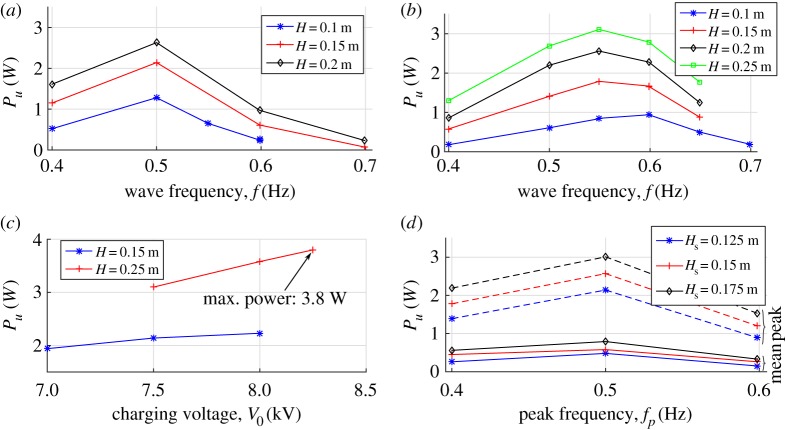
Experimental electrical power output of the Poly-A-OWC prototype in different operating conditions: (*a*) regular waves, *t*_0_ = 2 mm, *C*_*a*_ = 300 nF, *V*_0_ = 7.5 kV; (*b*) regular waves, *t*_0_ = 3 mm, *C*_*a*_ = 247 nF, *V*_0_ = 9 kV; (*c*) regular waves, *t*_0_ = 2 mm, *C*_*a*_ = 300 nF, *f* = 0.5 Hz; (*d*) irregular waves, *t*_0_ = 2 mm, *C*_*a*_ = 300 nF, *V*_0_ = 7.5 kV. (Online version in colour.)

Figure 8*a*,*b* shows the estimated power in the presence of different regular wave sea states, assuming two different layouts for the CD-DEG and different control parameters for the control electronics. In particular, figure 8*a* refers to a thinner membrane, while figure 8*b* refers to a thicker membrane, featuring a larger amount of active dielectric material. Owing to its larger stiffness, the thicker CD-DEG was tested with maximum wave heights of *H* = 0.25 m (as opposed to *H* = 0.2 m for the thinner DEG). In the plots, the power is estimated from the average of $W_{u}$ over the different cycles, and from the wave frequency, *f*. The first cycle after activation has been removed from the computation as it generally features different DEG oscillation amplitude with respect to the steady state response. Considering that the CD-DEG performs two generation cycles per period (one for upward and one for downward deformation), the following equality holds: $P_{u}=2W_{u}f$.

The electrical power is maximum at the design natural frequency (namely, *f* = 0.5 Hz). The power output surpasses 2 W in the case with thinner DEG, and 3 W in the case with thicker DEG, corresponding to roughly 300–440 kW at a scale 30 times larger, or 70–100 kW at a scale 20 times larger. This result is extremely encouraging from a wave energy application perspective, considering that it has been obtained with a non-optimal and rather dissipative material. It is expected that with purposely developed dielectric elastomers, performance would be further enhanced.

With the aim of measuring the peak power performance of the DEG prototype, a set of tests at constant excitation frequency equal to the prototype natural frequency (0.5 Hz) have been carried out. With reference to a DEG with thickness *t*_0_ = 2 mm, figure 8*c* shows the power output obtained with increasing values of the charging voltage *V*_0_ and the wave height *H*. A maximum power output of 3.8 W (140–560 kW full-scale equivalent) has been measured in tests with wave height *H* = 0.25 m and charging voltage *V*_0_ = 8.25 kV. From a DEG technology perspective, this result represents an important step-forward towards scaling-up, as the implemented power output target is significantly larger than that of typical DEG prototypes described in literature [33,34].

In panchromatic waves, the mean power *P*_*u*_ is computed as the sum of the electrical energy generated in the different cycles divided by the time duration of the generation test. Figure 8*d* shows the average power output for a CD-DEG sample over a set of irregular sea states. The power output is maximum when the peak frequency of the spectrum equals the natural frequency of the system (i.e. 0.5 Hz), although its dependence on the frequency is weaker than in monochromatic waves. Mean power of up to 1 W was successfully generated.

## 7. Model validation

In this section, we validate the nonlinear Poly-A-OWC numerical model against experimental results. Validation has been carried out starting from the validation of the hydrodynamic model of the collector, then including the electro-mechanical model of the DEG. Numerical results were obtained by implementing the presented set of equations in a Matlab & Simulink environment.

As regards the hydrodynamic model, a slightly more general version of the model presented in §3a has been employed in the validation procedure. Owing to large water column oscillations, indeed, the maximum downward displacements of the water column surface were larger than the distance between the SWL and the top section of the CD duct in certain tests. To account for consequent cross-section variations in the water column free surface, an extended version of the hydrodynamic model has been set-up, as described in the electronic supplementary material.

### (a) Hydrodynamic model validation

The collector hydrodynamic model has been validated against regular wave tests results on the open collector (with no DEG and water column free surface contacting the atmosphere), i.e. using the measured water column displacement, *z*. The comparison between experiment and model results is performed comparing the steady-state oscillation amplitudes of *z* in different sea states.

The model uses the collector geometrical dimensions and the wave parameters as the inputs, and employs the analytical equations presented in §3a to calculate the various dynamical coefficients. The viscous coefficient *K*_*v*_, which is the only uncertain parameter not known *a priori*, was preliminary calibrated. The calibration was carried out using the datasets relative to *H* = 0.15 m and different frequencies, identifying a value of *K*_*v*_ that minimizes the mean difference between model and experimental steady-state oscillation amplitudes. The selected value is *K*_*v*_ = 6.5. The details and outcome of the calibration procedure are described in the electronic supplementary material.

**Figure 9.**
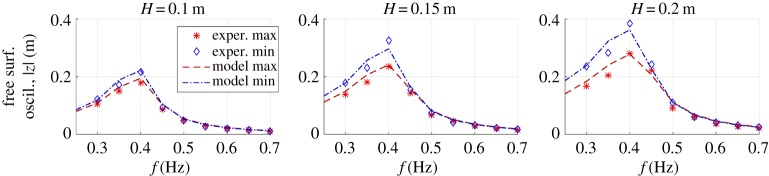
Comparison of experimental and model steady-state free surface oscillation amplitudes (maxima and minima of the profile, in magnitude) at different wave frequencies. Different sub-plots are for different wave heights. Markers indicate the experimental points, lines indicate the model points. (Online version in colour.)

Upon model calibration, model predictions were compared with experimental results considering a wider set of sea states than those used for the calibration. In figure 9, we compare experimental oscillation amplitudes with model predictions over a wide range of monochromatic sea states. Each sub-plot refers to a different wave height. In the plots, we report amplitudes of upward (*z* > 0) oscillations (red markers lines) and downward (*z* < 0) oscillations (blue markers and lines). Minima points (corresponding to downward oscillation amplitudes) are represented in the positive semi-plane to allow comparison of upward and downward oscillation amplitudes.

It can be noticed that due to nonlinearity (e.g. nonlinear inertia, quadratic forces), upward and downward oscillation amplitudes are different.

In terms of general trend, the model efficiently captures the following effects: value of the peak-amplitude frequency (resonance condition), wider oscillation amplitude for downward displacements.

The mean difference between model points and experimental points is 14.1% for upward oscillation amplitudes, and 8.8% for downward oscillation amplitudes (percentages are with respect to the experimental values). These values are the average throughout the different sea states. Overall, the mean difference between model oscillation amplitudes and experimental oscillation amplitudes is 11.5%.

Despite a large number of underlying assumptions, the proposed hydrodynamic model efficiently describes the axial-symmetric OWC dynamics, as it reproduces the main effects characterizing the collector dynamics, including the nonlinear ones.

### (b) Coupled Poly-A-OWC model validation

The previously validated hydrodynamic model was coupled to the CD-DEG electro-mechanical model described in §3b. Reference has been made to a CD-DEG sample with unstretched thickness *t*_0_ = 2 mm.

As in the hydrodynamic model case, validation was carried out comparing model predictions and experimental results in regular wave tests.

As regards the uncertain parameters, the same value of *K*_*v*_ used in the previous simulations was used, while a calibration of the membrane viscous damping coefficient, *B*_*h*_ (see equation (3.29)), was performed. Calibration was carried out using the dataset relative to *H* = 0.15 m and different frequencies, and using the steady-state oscillation amplitudes of the water column free surface, *z*, and the DEG tip height, *h*, which are dimensionally homogeneous, as the target variables. The datasets considered for calibration referred to Poly-A-OWC oscillations in the absence of electrical activation (idle operation). The value of *B*_*h*_ that minimizes the mean difference between model and experiments oscillation amplitudes is *B*_*h*_ = 250 kg (m^2^s)^−1^. Further details on the calibration are in the electronic supplementary material.

After calibration, estimated oscillation amplitudes of different physical variables were compared with experimental data. The comparison was carried out both in conditions of idle DEG operation (in the absence of electrical activation) and in fully-functioning conditions (in the presence of electrical activation with charging voltage *V*_0_ = 6 kV and in-parallel capacitance *C*_*a*_ = 394 nF).

**Figure 10.**
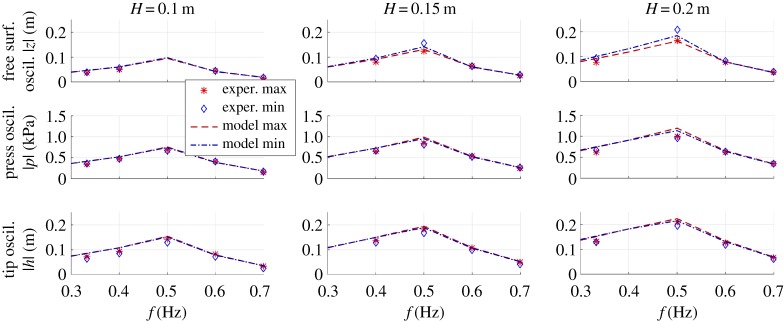
Comparison of experimental and model steady-state oscillation amplitudes (maxima and minima, in magnitude) of relevant physical variables (*z*, *p*, *h*) at different wave frequencies and heights. Data refer to tests with electrical activation. Markers indicate the experimental points, lines indicate the model points. (Online version in colour.)

For exemplification purpose, with reference to the steady-state DEG operation in the presence of electrical activation, in figure 10 we report a comparison of model and experimental oscillation amplitudes of *z*, *p* and *h* in a variety of regular sea states. The same results in the absence of electrical activation (idle Poly-A-OWC operation) are available in the electronic supplementary material.

Interestingly, despite natural discrepancies in the numerical values, the model is very efficient in predicting whether upward oscillations feature larger or smaller amplitude than downward oscillations, in the different conditions.

In table 2, we resume the percentage discrepancies of model predicted oscillation amplitudes from the experimental values, for both the cases with idle and electrically active DEG membrane. The agreement of the model with the experiments is remarkable, as the mean discrepancy is below 10%. This value is better than that pertaining the purely hydrodynamic model, mainly because the water column oscillation amplitudes in the presence of the DEG are smaller than in the presence of atmospheric air chamber.

**Table 2.** Mean percentage difference between model and experimental steady-state oscillation amplitudes over the different sea states. Percentages are with respect to the experimental values. Distinction is made between maxima and minima of the time-profiles.

	electrically inactive		electrically active	
	maxima	minima	maxima	minima
free surf. osc. ampl., |*z*| (%)	8.9	7.5	9.7	8.6
press. osc. ampl., |*p*| (%)	9.1	6.3	10.3	8.6
tip osc. ampl., |*h*| (%)	15.0	7.9	7.8	17.4

In conclusion, the presented validation procedure confirms that the proposed models capture the main features of the Poly-A-OWC dynamics, while requiring modest calibration (which can be performed using restricted experimental datasets).

Refinements of the models might include an improvement of the hydrodynamic parameters computation, and integration of the DEG model with accurate formulations for visco-elastic effects and electrical losses (e.g. leakage currents through the dielectric, electrodes resistivity).

Further results on hydrodynamic model validation are provided in the electronic supplementary material.

## 8. Conclusion

This paper presents a multi-physics model and validation of a new type of WEC equipped with a deformable polymeric PTO system, consisting of a DEG. DEGs are electrostatic devices that exploit variable capacitance to provide direct conversion of mechanical power into electrical power, while rejecting rigid, corrosion-sensitive and expensive components present in traditional electro-magnetic PTO systems.

The WEC under investigation, referred to as Poly-A-OWC, is an OWC equipped with an axial-symmetric collector holding a CD-DEG at its top. The peculiar U-shape of the collector is aimed at increasing the hydrodynamic inertia of the system and matching a target wave frequency with the device natural frequency.

An integrated model is proposed which includes a hydrodynamic sub-model for the OWC and an electro-elastic model for the CD-DEG PTO. Despite accounting for relevant nonlinear contributions, the model is built upon analytical equations and enables computationally-inexpensive simulation of the device dynamics, particularly suitable at design level.

Based on the proposed model, a small-scale Poly-A-OWC prototype (with a scale of approximately 1/20–1/30) has been designed and tested in a wave-tank. An extensive experimental campaign has been performed. The device has been tested in regular and irregular artificially generated waves. Tests provided an in-depth insight on the effect of the CD-DEG on the dynamical response of the system, and allowed the validation of the established integrated design procedure. Results of the validation procedure show that, despite the numerous assumptions, the model accurately describes the various phenomena involved in the Poly-A-OWC dynamics, including those related to nonlinear effects, and is thus an appropriate tool for design, new controllers testing, and concept evaluations.

Generation tests, in the presence of an electrical activation of the CD-DEG, were also performed. An electrical power output of a few Watts (equivalent to up to a few hundreds kilowatts at full-scale) has been observed both in regular and irregular waves, using different parameters for the CD-DEG, the conditioning electronics and the electrical control variables. Besides providing encouraging results for possible larger-scale energy harvesting applications, these results also represent a relevant scaling-up step for DEG technology. In the light of promising results achieved to date, further fundamental steps are required in order for DEGs to become a viable technology for wave energy PTO systems. These include: the synthesis of advanced dielectric materials, with enhanced dielectric strength and relative permittivity, capable to reliably withstanding millions of activation cycles at large electric fields; the development of scalable manufacturing processes for high-quality defect-free dielectric elastomer membranes with dimensions consistent with the wave energy application scale.

## Supplementary Material

Supplementary material on theory and experiments

## Supplementary Material

Video of the experimental sessions

## Data accessibility

Experimental timeseries are available upon request to the authors. Electronic supplementary material, consisting of additional text and video files, is available for this article.

## References

[1] Gunn K, Stock-Williams C 2012 Quantifying the global wave power resource. Renew. Energy 44, 296–304. (10.1016/j.renene.2012.01.101)

[2] Pecher A, Kofoed JP 2016 Handbook of ocean wave energy. Berlin, Germany: Springer.

[3] Falcão A 2010 Wave energy utilization: a review of the technologies. Renew. Sust. Energy Rev. 14, 899–918. (10.1016/j.rser.2009.11.003)

[4] Falcão AF, Henriques JC 2016 Oscillating-water-column wave energy converters and air turbines: a review. Renew. Energy 85, 1391–1424. (10.1016/j.renene.2015.07.086)

[5] Falcão AF, Henriques JC, Gato LM 2018 Self-rectifying air turbines for wave energy conversion: a comparative analysis. Renew. Sust. Energy Rev. 91, 1231–1241. (10.1016/j.rser.2018.04.019)

[6] Kornbluh RD, Pelrine R, Prahlad H, Wong-Foy A, McCoy B, Kim S, Eckerle J, Low T 2012 From boots to buoys: promises and challenges of dielectric elastomer energy harvesting. In *Electroactivity in polymeric materials* (ed. L Rasmussen), pp. 67–93. Berlin, Germany: Springer.

[7] Jean P, Wattez A, Ardoise G, Melis C, Van Kessel R, Fourmon A, Barrabino E, Heemskerk J, Queau J 2012 Standing wave tube electro active polymer wave energy converter. In *SPIE smart structures and materials+ nondestructive evaluation and health monitoring* (ed. Y Bar-Cohen). Bellingham, WA: International Society for Optics and Photonics.

[8] Moretti G, Fontana M, Vertechy R 2015 Parallelogram-shaped dielectric elastomer generators: Analytical model and experimental validation. J. Intell. Mater. Syst. Struct. 26, 740–751. (10.1177/1045389X14563861)

[9] Moretti G, Fontana M, Vertechy R 2015 Model-based design and optimization of a dielectric elastomer power take-off for oscillating wave surge energy converters. Meccanica 50, 2797–2813. (10.1007/s11012-015-0235-8)

[10] Kaltseis R, *et al.* 2014 Natural rubber for sustainable high-power electrical energy generation. RSC Adv. 4, 27 905–27 913. (10.1039/C4RA03090G)

[11] Moretti G, Papini GPR, Righi M, Forehand D, Ingram D, Vertechy R, Fontana M 2018 Resonant wave energy harvester based on dielectric elastomer generator. Smart Mater. Struct. 27, 035015 (10.1088/1361-665X/aaab1e)

[12] Rosati Papini GP, Moretti G, Vertechy R, Fontana M 2018 Control of an oscillating water column wave energy converter based on dielectric elastomer generator. Nonlinear Dyn. 92, 181–202. (10.1007/s11071-018-4048-x)

[13] Suo Z 2010 Theory of dielectric elastomers. Acta Mech. Solida Sin. 23, 549–578. (10.1016/S0894-9166(11)60004-9)

[14] Pelrine R, Kornbluh RD, Eckerle J, Jeuck P, Oh S, Pei Q, Stanford S 2001 Dielectric elastomers: generator mode fundamentals and applications. In *SPIE's 8th Annual Int. Symposium on Smart Structures and Materials, San Diego, CA*, pp. 148–156. International Society for Optics and Photonics.

[15] Moretti G, Righi M, Vertechy R, Fontana M 2017 Fabrication and test of an inflated circular diaphragm dielectric elastomer generator based on PDMS rubber composite. Polymers 9, 283 (10.3390/polym9070283) (10.3390/polym9070283) PMC6432430 30970961

[16] Falnes J 2002 Ocean waves, oscillating systems: linear interactions including wave-energy extraction. Cambridge, UK: Cambridge University Press.

[17] Boccotti P 2007 Caisson breakwaters embodying an OWC with a small opening - part I: theory. Ocean Eng. 34, 806–819. (10.1016/j.oceaneng.2006.04.006)

[18] Malara G, Gomes RPF, Arena F, Henriques JCC, Gato LMC, Falcão AFO 2017 The influence of three-dimensional effects on the performance of U-type oscillating water column wave energy harvesters. Renewable Energy J. 111, 506–522.

[19] Henriques J, Portillo J, Gato L, Gomes R, Ferreira D, Falcao A 2016 Design of oscillating-water-column wave energy converters with an application to self-powered sensor buoys. Energy 112, 852–867. (10.1016/j.energy.2016.06.054)

[20] Alves M 2012 Numerical simulation of the dynamics of point absorber wave energy converters using frequency and time domain approaches. PhD thesis, Universidade Técnica de Lisboa.

[21] Vertechy R, Rosati GPP, Fontana M 2015 Reduced model and application of inflating circular diaphragm dielectric elastomer generators for wave energy harvesting. J. Vib. Acoust. 137, 011016-1–011016-9. (10.1115/1.4028508)

[22] Dorfmann L, Ogden RW 2014 Nonlinear theory of electroelastic and magnetoelastic interactions. Berlin, Germany: Springer.

[23] Viuff TH, Andersen MT, Kramer M, Jakobsen MM 2013 Excitation forces on point absorbers exposed to high order non-linear waves. In *10th Ewtec 2013 European Wave and Tidal Energy Conf. Series, Aalborg, Denmark, 2–5 September*. Technical Committee of the European Wave and Tidal Energy Conference.

[24] McCormick ME 1973 Ocean engineering wave mechanics. New York, NY: Wiley.

[25] Yu Z, Falnes J 1995 State-space modelling of a vertical cylinder in heave. Appl. Ocean Res. 17, 265–275. (10.1016/0141-1187(96)00002-8)

[26] Huang J, Shian S, Diebold RM, Suo Z, Clarke DR 2012 The thickness and stretch dependence of the electrical breakdown strength of an acrylic dielectric elastomer. Appl. Phys. Lett. 101, 122905 (10.1063/1.4754549)

[27] Holzapfel GA 2000 Nonlinear solid mechanics - a continuun approach for engineering, vol. 24 Chichester, UK: Wiley.

[28] Foo CC, Cai S, Koh SJA, Bauer S, Suo Z 2012 Model of dissipative dielectric elastomers. J. Appl. Phys. 111, 034102 (10.1063/1.3680878)

[29] Ask A, Menzel A, Ristinmaa M 2012 Phenomenological modeling of viscous electrostrictive polymers. Int. J. Non-Linear Mech. 47, 156–165. (10.1016/j.ijnonlinmec.2011.03.020)

[30] Ingram D, Wallace R, Robinson A, Bryden I 2014 The design and commissioning of the first, circular, combined current and wave test basin. *Flow3d. com*.

[31] Falcão AF, Henriques JC 2014 Model-prototype similarity of oscillating-water-column wave energy converters. Int. J. Mar. Energy 6, 18–34. (10.1016/j.ijome.2014.05.002)

[32] Keplinger C, Li T, Baumgartner R, Suo Z, Bauer S 2012 Harnessing snap-through instability in soft dielectrics to achieve giant voltage-triggered deformation. Soft Matter 8, 285–288. (10.1039/C1SM06736B)

[33] Kaltseis R, Keplinger C, Baumgartner R, Kaltenbrunner M, Li T, Mächler P, Schwödiauer R, Suo Z, Bauer S 2011 Method for measuring energy generation and efficiency of dielectric elastomer generators. Appl. Phys. Lett. 99, 162904 (10.1063/1.3653239) (10.1063/1.3653239)

[34] Shian S, Huang J, Zhu S, Clarke DR 2014 Optimizing the electrical energy conversion cycle of dielectric elastomer generators. Adv. Mater. 26, 6617–6621. (10.1002/adma.v26.38) 25113278

[35] Koh SJA, Keplinger C, Li T, Bauer S, Suo Z 2011 Dielectric elastomer generators: how much energy can be converted?. IEEE/ASME Trans. Mechatron. 16, 33–41. (10.1109/TMECH.2010.2089635)

