# Supplementary material for: Modelling and testing of a wave energy converter based on dielectric elastomer generators
Source: Proc Math Phys Eng Sci. 2019 Feb 13;475(2222):20180566. doi: 10.1098/rspa.2018.0566 (PMC6405449; doi:10.1098/rspa.2018.0566)
Supplement: Supplementary material on theory and experiments [file rspa20180566supp1.pdf]

Supplementary material - "Modelling and testing of a wave energy converter based on dielectric elastomer generators"

Giacomo Moretti[1], Gastone Pietro Rosati Papini[1], Luca Daniele[1], David Forehand[2], David Ingram[2], Rocco Vertechy[3], and Marco Fontana[4]

[1]TeCip Institute, Scuola Superiore Sant'Anna, Pisa, Italy
[2]Institute for Energy Systems, The University of Edinburgh, UK
[3]Department of Industrial Engineering, University of Bologna, Italy
[4]Department of Industrial Engineering, University of Trento, Italy

This document is provided as supplementary material of the paper "Modelling and testing of a wave energy converter based on dielectric elastomer generators". Notice that references numbering recalls the enumeration provided in the main paper.

S1 Supplementary information on the hydrodynamic model

In this section, we discuss some mathematical details regarding the analytical models described in Section 3, including the detailed calculation of the wave excitation force and we provide a validation by comparison of the analytical hydrodynamic coefficients of the U-OWC collector (see Section 3(a)) and the numerical coefficients obtained with a BEM software (WAMIT), using reference collector dimensions equal to those of the experimental prototype investigated in the paper. Furthermore, an extended formulation of the model that holds in case the water column free surface intersects the CD duct variable cross section is provided.

S1.1 On the calculation of the wave excitation force

The wave excitation pressure, in equation (3.11), is calculated by averaging the pressure induced by an undisturbed wave over the inlet section of the U-OWC collector. Such a calculation is performed analytically as described in the following.

The pressure p_{uw} due to an undisturbed sinusoidal wave (namely, a regular wave) with height H and angular frequency ω in a generic point $(\xi - \eta - \zeta)$ of the sea water domain has the following expression [24]:

$$p_{uw} = \frac{\rho_w g H}{2} \frac{\cosh(k_w(h_w + \zeta))}{\cosh(k_w h_w)} \cos(k_w \xi - \omega \tau) \tag{S1}$$

with the symbols defined is Section 3(a).
The inlet section of the collector is a horizontal planar ring (comprised between the inner and the outer cylindrical shells), hereafter denoted by $\mathcal{R}$, that can be mathematically expressed as the following set of points (see also Figure 2a):

$$\mathcal{R} = \left\{(\xi, \eta, \zeta) : r_i^2 \leq \xi^2 + \eta^2 \leq r_o^2 \ \wedge \ \zeta = -a\right\}. \tag{S2}$$

Neglecting diffraction effects, the mean excitation pressure reads as follows:

$$\begin{aligned} p_e &= \frac{1}{\pi(r_o^2 - r_i^2)} \iint_{\mathcal{R}} p_{uw} \mathrm{d}\xi \mathrm{d}\eta = \\ &= \frac{\rho_w g H}{2\pi(r_o^2 - r_i^2)} \frac{\cosh(k_w(h_w - a))}{\cosh(k_w h_w)} \iint_{\mathcal{R}} \cos(k_w \xi - \omega \tau) \mathrm{d}\xi \mathrm{d}\eta = \\ &= \frac{\rho_w g H}{2\pi(r_o^2 - r_i^2)} \frac{\cosh(k_w(h_w - a))}{\cosh(k_w h_w)} \left(\cos(\omega\tau) \iint_{\mathcal{R}} \cos(k_w \xi) \mathrm{d}\xi \mathrm{d}\eta + \right. \\ &\left. + \sin(\omega\tau) \iint_{\mathcal{R}} \sin(k_w \xi) \mathrm{d}\xi \mathrm{d}\eta \right). \end{aligned} \tag{S3}$$

The second integral into parentheses on the right hand side of equation (S3) is null, because the integrand is an odd function of ξ and the integration domain $\mathcal{R}$ is symmetric with respect to the η axis. Expressing (ξ, η) in planar polar coordinates, $(\hat{r}, \hat{\theta})$, $\mathcal{R}$ can be expressed as follows:

$$\mathcal{R} = \left\{(\hat{r}, \hat{\theta}, \zeta) : r_i \leq \hat{r} \leq r_o \ \wedge \ 0 \leq \hat{\theta} \leq 2\pi \ \wedge \ \zeta = -a\right\}. \tag{S4}$$

Using the new coordinates in the integrals in equation (S3) and exploiting symmetries, p_e leads to the following expression:

$$
\begin{aligned}
p_e &= \frac{\rho_w g H}{2}\mathcal{L}(\omega)\frac{\cosh(k_w(h_w-a))}{\cosh(k_w h_w)}\cos(\omega\tau), \quad \text{with} \\
\mathcal{L}(\omega) &= \frac{4}{\pi(r_o^2-r_i^2)}\int_0^{\pi/2}\int_{r_i}^{r_o}\hat{r}\cos(k_w\hat{r}\cos\hat{\theta})\mathrm{d}\hat{r}\ \mathrm{d}\hat{\theta}
\end{aligned} \tag{S5}
$$

This expression is employed in the equations for the excitation force and excitation coefficient (equations (3.11) and (3.13) respectively).

S1.2 Comparison of analytical hydrodynamic coefficients with BEM

The hydrodynamic model presented in Section 3(a) relies on a significant number of assumptions/simplifications. For what concerns the contribution of the wave loads (excitation force, F_e, and radiation force, F_r, in equation (3.8)), the following assumptions have been introduced, to obtain fully-analytical expressions:

- The excitation force is calculated based on the undisturbed wave pressure neglecting diffraction (assumption that is often done for a point absorber). A wave excitation coefficient (depending on the frequency, ω) $\Gamma(\omega)$, is calculated accordingly. The radiation damping is calculated from $\Gamma(\omega)$, using Haskind relation (see equation (3.17)).

- The infinite-frequency asymptotic value of the system inertia is approximated with $M_z(z)$ (see equation (3.9)). $M_z(z)$ includes a dependence on z, but, coming from an integration on a finite water volume (coloured control volume in Figure 2a), it neglects the contribution of the water outside the U-OWC collector. An added mass contribution due to the surrounding water at lower frequencies is, in contrast, included in F_r through $\Delta M_{ad}(\omega)$ (see equations (3.16) and (3.18)). Moreover, the calculation of the hydrodynamic inertia uses the assumption of negligible horizontal velocity of the water flow, and the assumption that the water velocity is approximately null in proximity of the U-OWC collector bottom due to the inversion of the flow direction.

In order to evaluate the effect of those assumptions, in the following we present a comparison of coefficients calculated analytically with those calculated via BEM, for a reference U-OWC geometry. Frequency-domain radiation/diffraction BEM codes exist, which use linearised potential flow theory to solve for the wave motion about fixed and floating bodies. Using BEM, a set of linear hydrodynamic coefficients can be found (i.e., frequency-dependent coefficients, not depending on z, valid for small displacements from the equilibrium configuration, $z = 0$). Such parameters compare to the analytical ones, provided that the latter are computed at $z = 0$.

We make reference to the U-OWC geometry of the experimental prototype presented in the paper (see dimensions in Figure 3a), and we use the commercial BEM software WAMIT [S1] to compute relevant hydrodynamic parameters. The OWC device is modelled in WAMIT as a single body, constituted by the collector and by a lid (i.e., a horizontal flat surface) located in correspondence of the water column free surface (see also TEST 17b in [S1]). The translational degree-of-freedom of the water column free surface (z) is represented by defining a generalised degree-of-freedom [S2] on the lid, corresponding to the relative heave motion

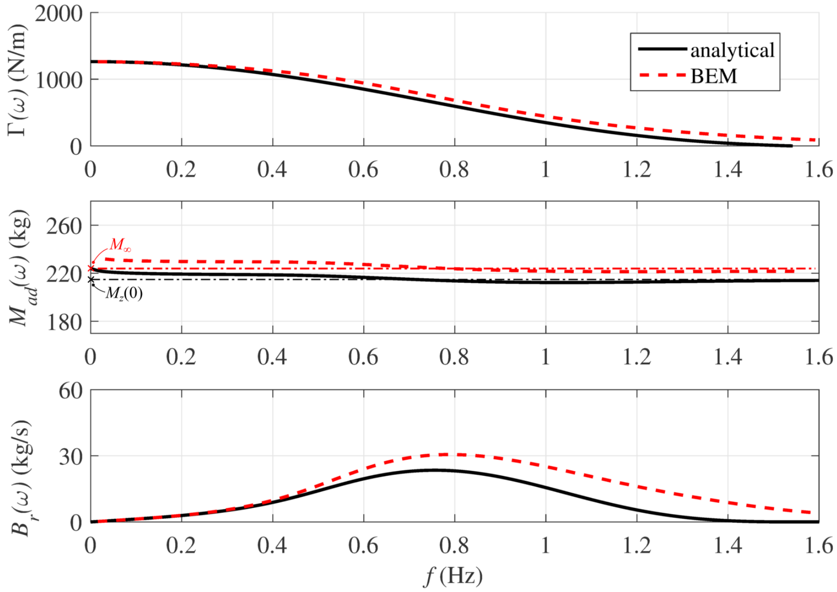

Figure S1: Comparison of hydrodynamic coefficients calculated via BEM (excitation coefficient $\Gamma(\omega)$, hydrodynamic added mass $M_{ad}(\omega)$, and radiation damping $B_r(\omega)$) with the corresponding analytical coefficients.

of the lid with respect to the collector. The body geometry is represented through surface meshes/panels. All of the body surfaces (collector walls and lid) are represented by dipole panels with null thickness. The solution for the hydrodynamic parameters is obtained using a lower order method [S1].

The output of the code execution is the following set of parameters: the linearised wave excitation coefficient, $\Gamma(\omega)$; the added mass, $M_{ad}(\omega)$, and its limit value (namely, infinite frequency added mass), M_∞; the radiation damping, $B_r(\omega)$. Notice that, owing to the definition of the OWC free surface through a thin lid, the added mass found by BEM corresponds to the total hydrodynamic inertia, and there is no further inertial contribution from the body.

In Figure S1 we present a comparison of the hydrodynamic parameters computed via BEM with the analytical ones. In particular, we compare: BEM and analytical excitation coefficients; the BEM added mass, $M_{ad}(\omega)$, with the total inertia calculated analytically at $z = 0$, namely, $M_z(0) + \Delta M_{ad}(\omega)$; and the radiation damping, B_r, either calculated from WAMIT or analytically.

The following can be observed:

- The analytical excitation coefficient is slightly underestimated, because diffraction has been neglected. This effect is accentuated at higher frequencies (larger than the operating frequencies of the prototype), in correspondence of which the device behaviour differs from that of a point absorber.

- The analytical added mass is slightly underestimated, mainly due to an underestimate of the infinite frequency added mass through $M_z(0)$ (neglecting the inertia of the fluid portion outside of the collector).

- The radiation damping calculated analytically using Haskind relation is overestimated, due to the underestimate in the excitation coefficient.

In practice, the difference between numerical and approximated analytical coefficients is considered sufficiently small for the intended purpose of the model . The parameter which is mostly affected by the assumptions of the analytical model is radiation damping.

Instead of using the above mentioned assumptions to analytically compute the hydrodynamic coefficients, an alternative approach consists in calculating the hydrodynamic parameters with a BEM code and analytically correct them in order to introduce a dependence on z, as suggested in [18]. However, this implies that the BEM calculation of the parameters (including geometric design of the mesh and BEM code execution) has to be re-run every time the U-OWC geometry is updated. It seems that the best option, at the design level, is to use a fully-analytical model, to be possibly refined with BEM-generated hydrodynamic coefficients once the final WEC geometry has been defined.

S1.3 Hydrodynamic model formulation in the presence of a variable cross-section water column

In some experimental tests on the Poly-U-OWC prototype with particularly large water column oscillations, the maximum downward displacement of the water column was found to go below the top section of the CD duct. In those conditions, some of the equations presented in Sect. 3(a) do no rigorously hold. In the following, we present an extension of the hydrodynamic model which accounts for such an effect.
For that purpose, we consider a U-OWC collector whose main water chamber has a variable radius cross-section, with local radius equal to $r(\zeta)$, as shown in Figure S2a. The cross-section radius in correspondence of the water column free surface is thus indicated with $r(z)$ (in general, different from r_i).
With these definitions, Eq. (3.1) takes the following form

$$\dot{m} = \rho_w \pi r^2(\zeta) v(\zeta) = \rho_w \pi \left(r_o^2 - r_i^2\right) v_i = \rho_w \pi r^2(z) \dot{z}. \tag{S6}$$

The various terms of the global energy balance take the following alternative expressions. The kinetic energy $\mathcal{E}_k$ is the sum of two terms: one owing to the fluid in the added mass duct, and one owing to the fluid volume within main water column:

$$\mathcal{E}_k = \pi \rho_w r^4(z) \left(\frac{b - a - c_s}{r_o^2 - r_i^2} + \int_{-b+c_s}^{z} \frac{\mathrm{d}\zeta}{r^2(\zeta)} \right) \frac{\dot{z}^2}{2}. \tag{S7}$$

The potential energy of the control volume (with zero-potential-energy set-point on the SWL) has the following expression:

$$\mathcal{E}_g = \mathcal{E}_{g,j} + \pi \rho_w g \int_{-b+c_s}^{z} r^2(\zeta) \zeta \mathrm{d}\zeta \tag{S8}$$

where $\mathcal{E}_{g,j}$ is the constant potential energy of water volume in the acceleration duct, while the second term is the potential energy of the water within the collector inner body.

The hydrodynamic viscous dissipated power $\dot{\mathcal{W}}_{vh}$ has the following expression:

$$\dot{\mathcal{W}}_{vh} = -\frac{\pi \rho_w K_v r^6(z)}{2(r_o^2 - r_i^2)^2} \dot{z}^2 |\dot{z}| \tag{S9}$$

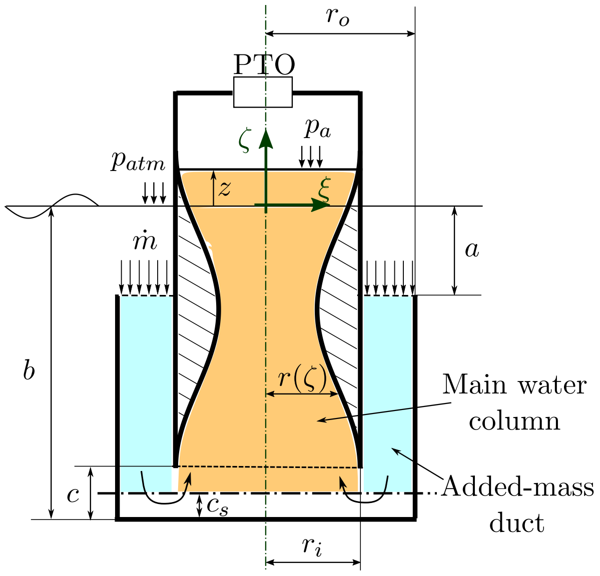

(a)

Figure S2: U-OWC geometry and dimensions in the presence of a variable cross section of the main water column chamber.

The mechanical power acting on the water column free surface reads as follows:

$$\dot{\mathcal{W}}_a = -\pi r^2(z) p_a \dot{z}. \tag{S10}$$

Finally, $\dot{\mathcal{W}}_{in}$ takes the following form:

$$\dot{\mathcal{W}}_{in} = \pi r^2(z)\dot{z}\left[p_{atm} + \frac{\rho_w r^4(z)}{(r_o^2 - r_i^2)^2}\frac{\dot{z}^2}{2} + p_e + p_r\right] \tag{S11}$$

Based on those terms, the water column equation of motion (Eq. (3.8)) is replaced by the following more general equation:

$$M_z(z)\ddot{z} = -C_v\dot{z}^2 - \pi\rho_w g r^2(z) z - \frac{\pi\rho_w K_v r^6(z)}{2(r_o^2 - r_i^2)^2}|\dot{z}|\dot{z} - \pi r^2(z) p + F_e + F_r, \tag{S12}$$

with the following differences in the expressions of the various hydrodynamic parameters.

- Generalised water column inertia:

$$M_z(z) = \pi\rho_w r^4(z)\left(\frac{b - a - c_s}{r_o^2 - r_i^2} + \int_{-b+c_s}^{z}\frac{\mathrm{d}\zeta}{r^2(\zeta)}\right) \tag{S13}$$

- Quadratic force coefficient:

$$C_v = 2\pi\rho_w r^2(z)\left\{\left[\frac{b - a - c_s}{r_o^2 - r_i^2} + \int_{-b+c_s}^{z}\frac{\mathrm{d}\zeta}{r^2(\zeta)}\right] r(z) r'(z) + \frac{1}{4} - \frac{r^4(z)}{4(r_o^2 - r_i^2)^2}\right\} \tag{S14}$$

  where $r'(z)$ is the derivative of $r(\zeta)$ calculated in z.

- Wave-induced forces:

$$
\begin{aligned}
&F_e(z,\tau) = \pi r^2(z) p_e = \pi r^2(z) \frac{\rho_w g H}{2} \mathcal{L}(\omega) \frac{\cosh(k_w(h_w - a))}{\cosh(k_w h_w)} \cos(\omega\tau), \\
&F_r(z,\tau) = \pi r^2(z) p_r
\end{aligned}
\tag{S15}
$$

It is worth noticing that, compared to the original formulation with constant cross-section of the water column free surface, here the excitation and radiation forces generally depend on z. In particular, based on Eq. (S15), a nonlinear excitation coefficient can be defined:

$$
\Gamma_z(\omega, z) = \pi r^2(z) \rho_w g \mathcal{L}(\omega) \frac{\cosh(k_w(h_w - a))}{\cosh(k_w h_w)}. \tag{S16}
$$

The calculation of the nonlinear radiated wave load is, in contrast, rather complicated, as the radiation potential is a function of z and $\dot{z}$ and it is not known a priori. It is then convenient, at least for this contribution, to make use of a linear formulation as in the main paper. In practice, F_r can be expressed as a convolution integral as per Eq. (3.15), with a convolution kernel featuring the frequency-domain expression given by Eq. (3.16). Therefore, the frequency-dependent radiation damping coefficient $B_r(\omega)$ can be calculated replacing $\Gamma(\omega)$ in Eq. (3.17) with $\Gamma_z(\omega, 0)$. Finally, the frequency-dependent added-mass coefficient can be calculated from such a damping coefficient through Eq. (3.18).

In addition, in the present case, as the air chamber volume variation due to the water column displacement becomes a non-trivial function of z (and of the DEG tip displacement, h), Eq. (3.32) takes the following form:

$$
p_{atm} V_{a0}^{\gamma} = (p + p_{atm}) \left(V_{a0} - \int_0^z \pi r^2(\zeta) \mathrm{d}\zeta + \Omega_c(h) \right)^{\gamma}. \tag{S17}
$$

S2 Video analysis of the membrane tip displacement

This section describes the video post-processing procedure for the CD-DEG's tip elevation identification. The procedure relies on a video analysis performed on the frames of a high-speed camera, that was secured to the tank gantry during the experiments. The camera was used to film the CD-DEG deformations at a speed of 100 frames per second. Figure S3 shows a schematic, in which the relative positions of the high speed camera, the Poly-U-OWC and of a white background panel used to increase the quality of the video identification are shown with respect to the tank.

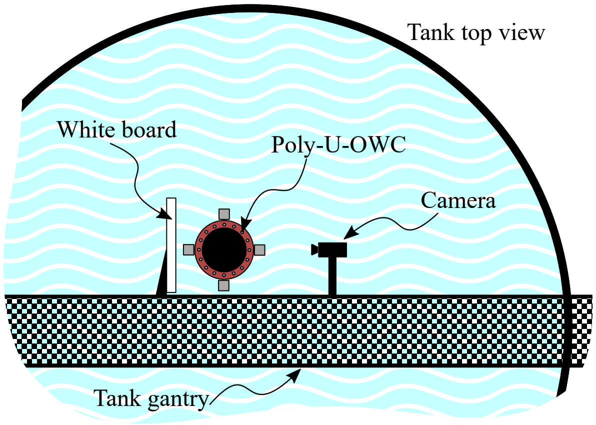

Figure S3: Position of the camera in Flowave wave-tank.

Figure S4 shows the main steps of the procedure to obtain the time-series of the tip elevation of the membrane. The video identification procedure is divided in two main parts.

The first part consists of a processing phase of the video frames aimed at increasing the contrast between the membrane shape and the background. In the figure, this part is represented by Steps a to g. The core of this procedure, represented by `Step g`, is the computation of the image difference between the current video frame and a reference frame taken from the video when the membrane is flat.
The algorithm relative to the second part of the procedure takes the membrane contrasted image and performs a routine in order to obtain the numerical value of the membrane tip elevation (`Step h` and `Step i`).
Since the membrane tip elevation taken from the video elaboration of the high-speed camera frames is measured in pixels, the tip elevation values in metres are obtained applying that is deduced from the analysis of the video frames assuming the diameter of the membrane as reference dimension.

Specifically, a simplified code relative to the first part of the identification procedure is provided in the code boxes in Figure S5 and Figure S6.
In particular, the code box Figure S5 shows the main steps to obtain a series of base frames, identified as `IB` in the code box. The base frames are continuously updated due to illumination issues, indeed the frequency of the lamps is 50 Hz and it is close to the camera frequency, thus generating a light intensity fluctuation. To solve the problem, the first base frame is taken as the first frame of the video, in correspondence of which the membrane is flat as it has not started to move yet. The successive base frames are taken each time the membrane goes

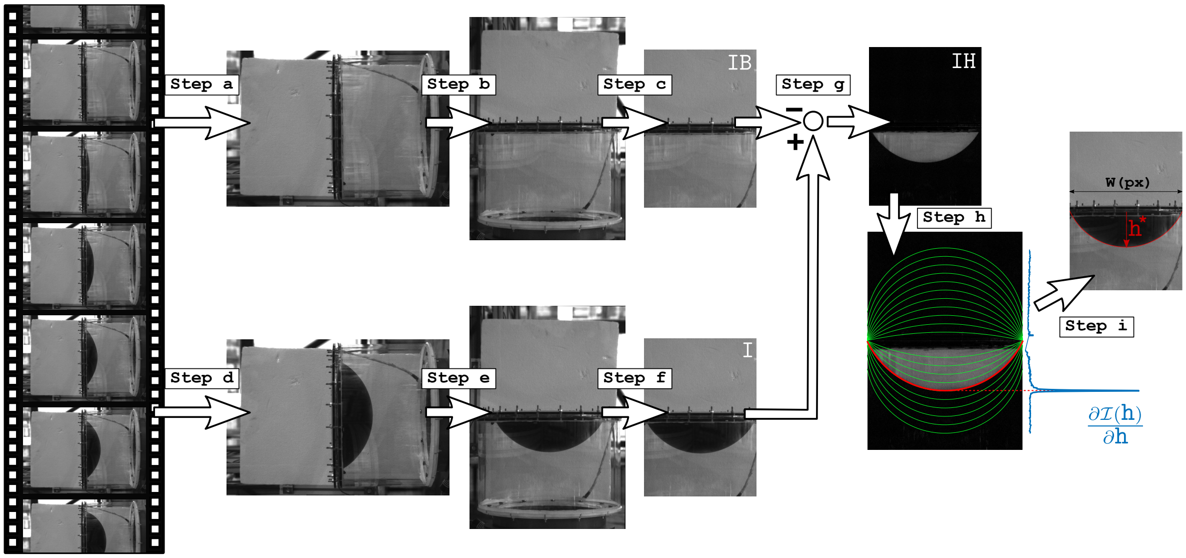

Figure S4: CD-DEG tip identification procedure.

behind the horizontal support flanges, i.e., when it is not visible in the video and its position is close to the flat configuration. This solution rejects the light intensity fluctuation, avoiding the base frame light intensity to be different from that of the video frame under investigation. When the algorithm detects that the frame under investigation is a base frame, the procedure reads as follow (see Figure S5):

`Step a` Reading of the `i`-th frame, `IB`, from the video (coded as `videoObj`).

`Step b` The image is first rotated by 90°, then of a little amount equal to `angle` to compensate the little error of positioning/orientation of the camera. The camera has been oriented in such a way that the longer side of its field of view is aligned with the vertical axis of the CD-DEG.

`Step c` The image is cropped, to reduce its dimension and accelerate the video analysis. The size of the cropping area is `W`×`H`. Variable `mlx` identifies the horizontal position of the vertical symmetry plane of the membrane. Variable `mly` identifies the vertical position of the membrane flat configuration.

In case the current frame is not identified as a base frame, Figure S6 shows the procedure to obtain the image with contrasted membrane shape, `IH`, from the current image, `I`. Steps `d`, `e`, `f` are equal to those previously described in the base frame identification procedure. `Step g` is the image difference between the last base frame, `IB`, and the frame under investigation, `I`.

`IH` is a grey scale image (i.e., it associates a value between 0 and 1 to each pixel of the image), and it is processed to obtain the tip elevation, h, measured in metre. Indicating with x and y the two coordinates of the image, $\mathcal{I}(\mathtt{h})$ is the integral of the gradient of `IH` along the y axis over an image portion, $\Omega(\mathtt{h})$. $\Omega(\mathtt{h})$ is a circular segment with height (sagitta) `h`. $I(\mathtt{h})$ is mathematically defined as follows:

Video analysis script for the base frame

```
IB = read(videoObj, i);                          #Step a
IB = imrotate(IB,-90);                           #Step b
IB = imrotate(IB,angle,'bilinear','crop');
IB = imcrop(IB,[mlx-W/2,mly-H/2,W,H]);           #Step c
```

Figure S5: The main steps to obtain the base frame, with the relative script main phases.

Video analysis script for the current frame

```
I = read(videoObj, i);                           #Step d
I = imrotate(I,90);                              #Step e
I = imrotate(I,angle,'bilinear','crop');
I = imcrop(I,[mlx-W/2,mly-H/2,W,H]);             #Step f
IH = imabsdiff(I,IB);                            #Step g
```

Figure S6: The main steps for the image filtering, with the relative script main phases.

$$\mathcal{I}(\mathtt{h}) = \int_{\Omega(\mathtt{h})} \left| \frac{\partial \mathtt{IH}(x,y)}{\partial y} \right| \mathrm{d}x \mathrm{d}y \tag{S18}$$

The magnitude of the gradient of $\mathtt{IH}(x, y)$ along y is calculated in order to have a image, that is mainly black where the variation of the colour is smooth and it is white where the variation is sudden.

The magnitude of the gradient of $\mathtt{IH}(x, y)$ along y is nearly null in the image portions where the grey scale is homogeneous, while it is maximum in correspondence of the border of the membrane cap, where the grey scale undergoes a sudden variation. Normalizing the gradient magnitude between 0 and 1 and plotting it as a grey scale image results in a black frame with the edge of the deformed membrane cap marked in white (see Figure S7).
As the membrane shape is very well approximated by that of a spherical cap, the mentioned integration on a circular segment domain is used to put in relation the configuration of the membrane with its tip elevation. In particular, by increasing $\mathtt{h}$, the value of the integral $\mathcal{I}(\mathtt{h})$ mildly increases as long as the considered circular segment does not contain the membrane border (where the gradient is maximum), and it features a sudden increase in correspondence of the value of $\mathtt{h}$ that matches the actual membrane shape.

Figure S7 shows the details of this procedure for the same video frame shown on the bottom right corner of Figure S4. Figure S7 depicts the magnitude of the vertical gradient of $\mathtt{IH}(x, y)$ (that is a mainly black image with only the strip of the membrane's contour in white) on the left, and the profile of $I(\mathtt{h})$ on the right. The profile of the gradient of $\mathcal{I}(\mathtt{h})$ with respect to $\mathtt{h}$ is sown in Figure S4 (`Step h`). The value of $\mathtt{h}$ in which the function $\partial\mathcal{I}(\mathtt{h})/\partial\mathtt{h}$

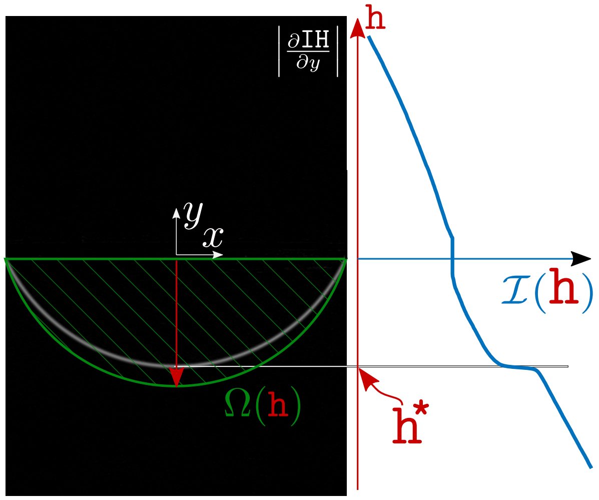

Figure S7: Detail of `Step h`. The grey scale image on the background represents the normalized magnitude of the gradient of `IH` along y, i.e., the white strip represents the points where the gradient is maximum, i.e., at the membrane border. The green hatching represents a generic circular segment with height `h`.

has a peak corresponds to the actual tip elevation of the membrane, $\mathtt{h}^*$, as shown by `Step i` in Figure S4.

The value of $\mathtt{h}^*$ measured in pixel is reported to metres using the scale factor `S` defined by the following equation:

$$\mathtt{S} = \frac{2e}{\mathtt{W}}, \tag{S19}$$

where `W` is the width of the image in pixel, equal to the width of the membrane, and e is the radius of the membrane in metre. Therefore, the tip height in metre is $h = \mathtt{S}\mathtt{h}^*$.

Based on the camera pixel dimensions and the employed video resolution, the uncertainty in the estimation of the CD-DEG tip height is less than 0.6 mm.

The described video processing procedure has been entirely implemented using MATLAB® scripts.

S3 On the calculation procedure of the generated electrical energy

The electrical energy generated by the CD-DEG in a cycle is assessed by indirect measurement using equation (6.1). As pointed out in Section 6(b), assuming a constant CD-DEG capacitance (C_A) during the charging phase (phase (2) in Figure 5b) is an approximation, since such a capacitance would slightly vary owing to the DEG deformation induced by the electrical stresses that arise when an electric field is applied on the DE layers (the DEG undergoes a further little expansion when it is electrically charged).
In effect, conversion cycle OABO in Figure 5b should be more accurately represented as in Figure S8a, where the straight iso-capacitance line OA has been replaced by a bent curve (here, the slope of the curve has been exaggerated for exemplification purposes). Such a curve is made by a sequence of equilibrium states featuring variable (increasing) CD-DEG capacitance.

In the following, we show that approximating curve OA with a straight line (as in equation (6.1)) involves a negligible error in the generated energy calculation (lower than that naturally present on the measured variables).

With reference to the current CD-DEG layout described in Table 1, Figure S8b shows the limit curves relative to the different operating constraints (break-down, rupture, minimum capacitance, electro-mechanical stability), obtained with established CD-DEG electromechanical models [21]. For the sake of representation, it has been assumed that the breakdown electric field, E_{BD}, equals 66 MV/m in the unstretched state and it increases proportionally to $\lambda^{0.6}$ (where λ is given by equation (3.19))based on previous experiments on VHB 4905, while mechanical rupture is assumed to occur when λ reaches a value of $\lambda_u = 8$.

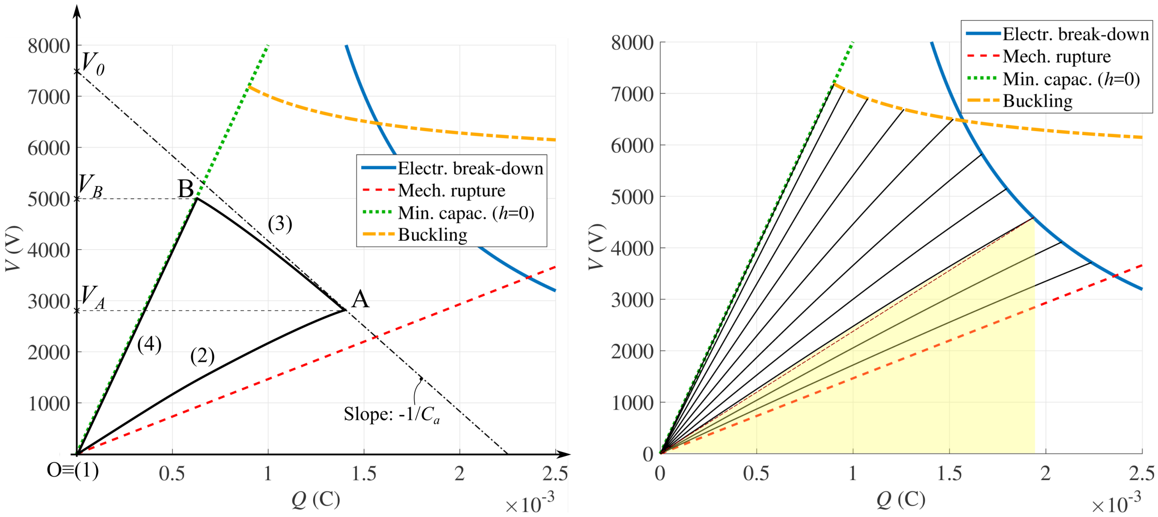

(a) (b)

Figure S8: (a) Operating constraints of the reference CD-DEG sample and example of real (non-ideal) conversion cycle (OABO) (b) Iso-z curves (solid black-lines), corresponding to different positions of the water surface and representing equilibrium states for the CD-DEG and the air chamber at different applied voltages.

A set of black solid lines are shown in the plot. Each line represents a charging curve,

corresponding to a different initial membrane deformation. Curve OA in Figure S8a ideally represents a portion of one of those curves.
Each of the curves is obtained as follows. An initial position, z, of the the water column is assumed, to which a certain deformation of the (inactive) CD-DEG is associated, using equations (3.29) and (3.32). It is then assumed that the CD-DEG is charged to a maximum voltage (set by either buckling or break-down condition). Since charging operation is very fast (approximately instantaneous), the position, z, of the water column during this operation does not change (owing to the water column inertia). The response of the air chamber and the CD-DEG is, on the contrary, quasi-static (i.e., their inertia is approximately negligible and, in this calculation, B_h is also neglected), thus, the equilibrium position of the membrane and the volume of the air chamber change as a result of a voltage-induced variation in the CD-DEG stresses.

Each black line corresponds to a constant value of z and is constituted by a set of equilibrium points for the CD-DEG and the air chamber at different intermediate voltages, in accordance with equations (3.32) and (3.29).

For each of the charging curves in Figure S8b, we have calculated the subtended area and compared it with that of the secant straight line passing through the axes origin and the intersection point of the curve with the break-down/buckling curve (see the example in Figure S8b). We found that, approximating the charging curves with straight lines leads to a maximum error of less than 1% in the calculation of the priming energy. This error becomes even lower, considering that, in practice, point A lies below (i.e., at lower voltage) the buckling/break-down curve (compare Figure S8a and Figure S8b).

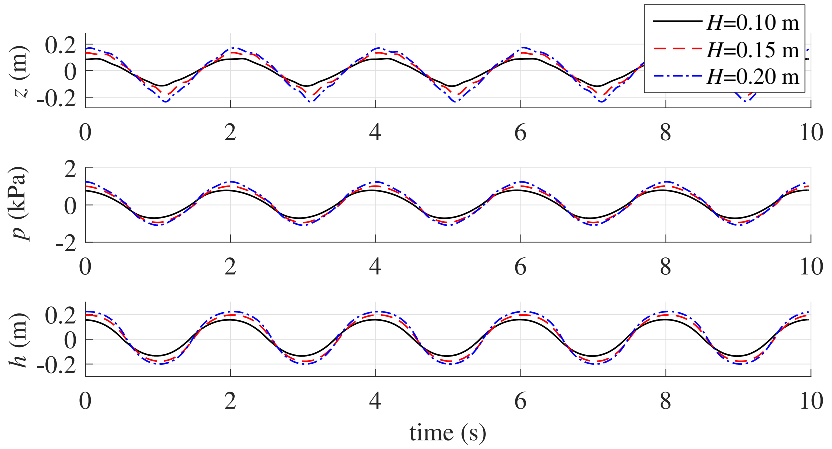

Figure S9: Experimental time-series of water column displacement (z), air chamber gauge pressure (p), and membrane tip elevation (h) in the presence of regular waves with frequency $f = 0.5$ Hz and different heights (H =0.10, 0.15 and 0.20 m).

S4 Experimental results

In the following, we provide a more detailed overview of experimental datasets beyond those presented in Section 6 of the main paper.

Figure 6 in the main paper compares the oscillation amplitude of some relevant physical variables in the presence of regular waves with same height and different frequencies. In order to assess the influence of the wave height on the dynamics, in Figure S9 we compare the oscillation amplitudes of the water column free surface, air pressure, and membrane tip height in the presence of a set of regular waves with frequency of 0.5 Hz. The plots refer to a test in the absence of electrical activation (idle device) and a CD-DEG with unstretched thickness $t_0 = 2$ mm. The plot shows that the increase in the oscillation amplitudes is greater when H varies from 10 cm to 15 cm than when it varies from 15 cm to 20 cm. This is an effect of the nonlinear DEG behaviour, which results in a saturation of deformation and displacements as the excitation forces increase. As pointed out in Section 6, this saturation also influences the trend of the device electrical power output (in the presence of electrical activation) which rises less than quadratically with the incident wave power (see Figures 8a and 8b).

As regards the electrical performance of the device in the presence of electrical activation, we hereby report other results from both regular and irregular wave tests beyond those shown in Figure 8 in the main paper.

In particular, Figure S10 compares the device electrical power output in the presence of a CD-DEG membrane with $t_0 = 2$ mm, with an in-parallel capacitor with $C_a = 394$ nF. Figures S10a and S10b refer to two different values of the charging voltage V_0 (5 and 6 kV respectively). A comparison of the two sub-plots show that the peak power output increases more than linearly with the increasing charging voltage, V_0.

As regards power generation tests in irregular waves, beside the tests described in Figure 8d, other tests were carried out using the same membrane asset. In particular, Figure S11a shows the instantaneous power time-series for a panchromatic wave with f_p =0.5 Hz and H_s =0.225 m. The instantaneous power is piece-wise constant and it is defined as the ratio of the energy generated in a certain cycle divided by the duration of that cycle, identified by

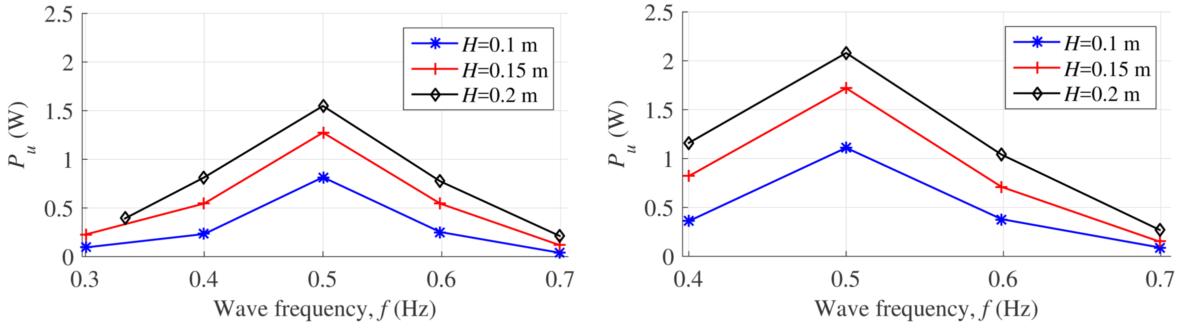

(a) (b)

Figure S10: Experimental electrical power output of the Poly-U-OWC prototype in different regular wave conditions. In the subplots, the CD-DEG has same thickness ($t_0 = 2$ mm), the in-parallel capacitance is $C_a = 394$ nF and the activation voltage is (a) $V_0 = 5$ kV, (b) $V_0 = 6$ kV respectively.

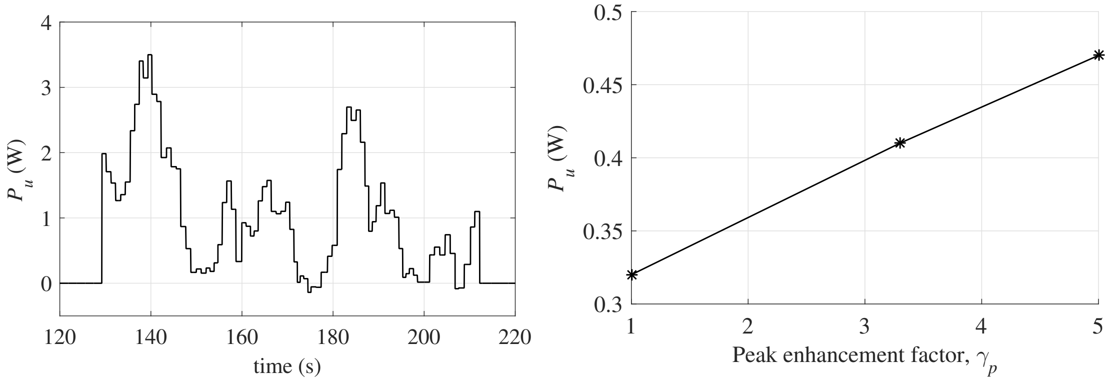

(a) (b)

Figure S11: (a) (b) Instantaneous power time-series for a panchromatic sea state featuring $f_p = 0.5$ Hz and $H_s = 0.225$ m. (b) Experimental mean power output of the U-OWC in panchromatic sea states featuring JONSWAP spectrum with $f_p = 0.5$ Hz and $H_s = 0.225$ m, and different values of the peak enhancement parameter, γ_p.

two successive apertures of switch S_2 (see Figure 5a). A mean power of 1.1 W and a peak power of 3.5 W were measured. The time-series shows that the controller is effective also in panchromatic waves, and the power output is positive practically everywhere throughout the considered time interval.

In Figure S11b, we compare mean power outputs of a CD-DEG operated in irregular waves featuring $H_s = 0.15$ m and $f_p = 0.5$ Hz (i.e., close to the Poly-OWC device natural frequency) and different values of the peak enhancement parameter, γ_p [2]. The mean power output increases with γ_p, i.e., when the deviance of the spectrum from the peak decreases, and the waves profile tends to a regular wave. In fact, in this case, increasing γ_p leads the prototype closer to the resonant monochromatic condition.

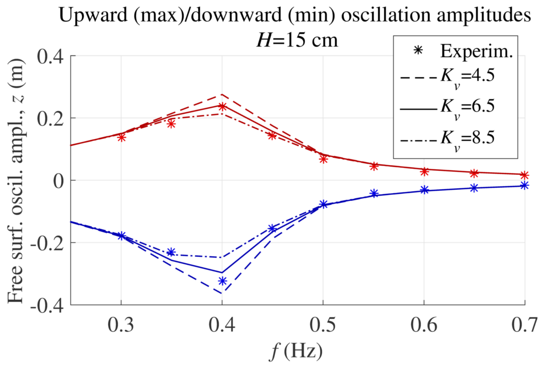

(a) Calibration of K_v

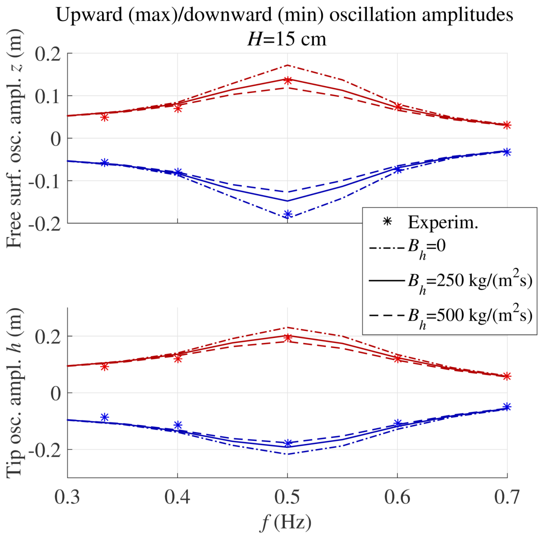

(b) Calibration of B_h

Figure S12: Model calibration. The plots show the steady-state upward (maxima) and downward (minima) oscillation amplitudes of some relevant physical variables in regular waves with same amplitude and different frequencies: (a) water column free surface oscillation amplitude with atmospheric air chamber, at different values of K_v; (b) water column and CD-DEG tip oscillation amplitudes in the presence of a DEG with $t_0 = 2$ mm (in the absence of electrical activation), at different values of B_h.

S5 Model validation

This section presents further results relative to the models validation procedure, in addition to those presented in Section 7 in the article.

S5.1 Model calibration

Figure S12 shows some results relative to the model calibration procedure. The only parameters that are not deduced from theoretical considerations and need a calibration are the dissipative parameters K_v and B_h, accounting for the hydrodynamic viscous losses (equation (3.5)) and membrane visco-elastic losses (equation (3.29)) respectively.

The hydrodynamic viscous coefficient is calibrated upon the experimental results collected on the open collector (with atmospheric air chamber, without the CD-DEG) in regular wave tests, in the presence of wave height $H = 0.15$ m. Time-domain simulations at different frequencies have been performed, using a set of values of K_v. For each simulation, the steady-state oscillating response is taken into account, and maxima/minima of the free surface oscillations above equilibrium ($z = 0$) are compared with experimental steady-state maxima/minima. The selected value of K_v is the one that minimizes the mean difference between experimental and model points (i.e., maxima/minima at the different frequencies). In Figure S12a, we report a comparison of the experimental oscillation amplitudes (with sign) and the model outputs at different values of K_v (a few values of the viscous coefficient are considered in the plot for better readability). The selected coefficient value is $K_v = 6.5$.

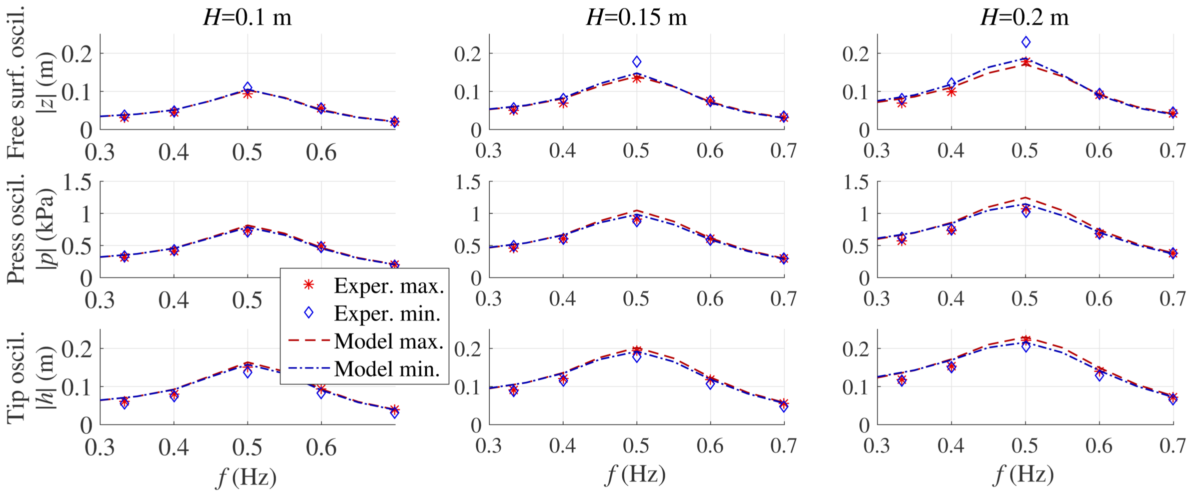

Figure S13: Comparison of experimental and model steady-state oscillation amplitudes (magnitude of the maxima and minima) of relevant physical variables (z, p, h) at different wave frequencies and heights. Data refer to tests on the idle DEG, without electric activation. Markers indicate the experimental points, lines indicate the model points.

In a similar way, calibration of B_h is carried out using regular wave datasets at constant wave height, $H = 0.15$ m. Consistently with the model validation results of the paper, the calibration has been carried out considering a CD-DEG with the same features considered in Section 7(b). Simulations at different wave frequencies are performed using a set of values for B_h. Variables z and h are chosen as the target for calibration, as they are dimensionally homogeneous. The steady-state extreme points (maxima/minima) in the model's time-series of z and h are then compared with the experimental results, making reference to idle oscillation of the systems, in the absence of electrical activation. A value of B_h is selected which minimizes the mean difference between the various model and experimental points. In Figure S12b, we present a comparison of the model and experimental extreme points (with sign) at different values of B_h (only three values of B_h are considered in the picture for graphical clarity).

S5.2 Idle (electrically inactive) Poly-U-OWC response validation

Figure 10 in the paper shows the steady-state oscillation amplitudes of z, p and h in regular sea states when the electrical controller is active.

In Figure S13, in contrast, we consider the steady-state oscillation amplitudes in the absence of electrical activation (as in the first part of the time-series in Figure 6). The plots show a fair agreement between model and experimental data, with discrepancies below 15 %, as shown in Table 2.

S5.3 Model and experimental time-series comparison

Model validation, in the paper, is carried out comparing the oscillation amplitudes (maxima and minima) of relevant physical variables in regular waves. However, it is also interesting to assess the model capability to punctually replicate the time-series of the relevant physical variables both in regular and irregular waves. To do so, regular wave simulations can be easily done by feeding the regular wave parameters (height, H, and period, T) into the model,

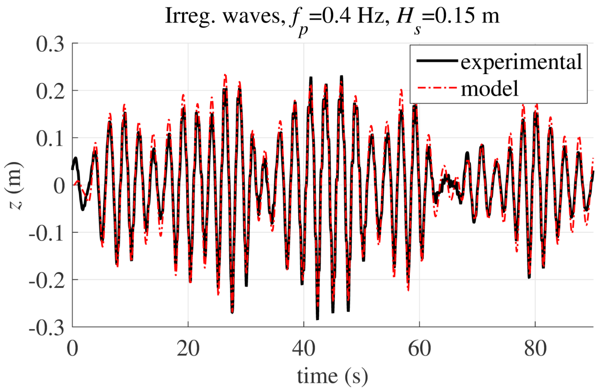

Figure S14: Comparison of model and experimental water column free surface displacement time-series, in the presence of an irregular incident wave, for the case with atmospheric OWC collector.

while simulations in irregular waves can be accomplished by decomposing the measured wave elevation (in the device far-field) into a finite superposition of harmonics using Fast Fourier Transform and then using those harmonics to generate the excitation force profile according to equation (3.14).

With reference to the purely hydrodynamic model and tests (in the absence of the DEG), Figure S14 shows a comparison of theoretical and experimental time-series of the water column elevation, z, in the presence of an irregular wave train (generated using a JONSWAP spectrum with peak enhancement factor of 3.3). The plot confirms the good agreement of the models with the experiments also in panchromatic waves. In particular, the model slightly overestimates the oscillation amplitude in smaller wave trains, while it exhibits better agreement (or slight underestimation) in larger waves, as already visible from regular waves results by comparison of the sub-plots in Figure 9.

With reference to electrical tests on the Poly-U-OWC prototype with CD-DEG (in the presence of electrical activation with charging voltage $V_0 = 6$ kV and in-parallel capacitance $C_a = 394$ nF), Figure S15 shows a comparison of model and experimental time-series for a set of three different tests (featuring $H = 0.15$ m and different frequencies) during which the DEG is electrically inactive in the first half of the test, and electrically controlled during the second part of the test. The plots show that, although the calibration has been performed based on the idle DEG operation, the model works effectively also in the presence of a voltage, indeed it is able to replicate the voltage profiles and the electrically-induced modifications in the other variables time-series quite accurately. Most importantly, the model captures the following effects, previously discussed in Section 6(a): pressure drop in correspondence of the quick DEG charging transient; increase/decrease in the steady-state oscillation amplitudes of z, p and h due to electric activation, depending on the wave frequency.

Finally, with reference to generation tests with in parallel capacitance $C_a = 300$ nF and a charging voltage of $V_0 = 7.5$ kV, in Figure S16a and b, we compare model and experimental panchromatic time-series of z, p, h and V. In general, matching the different time-series of such kind of experiments is not trivial, especially for what concerns the CD-DEG voltage, whose profile is very sensitive to relatively small errors on the DEG tip displacement (the CD-DEG capacitance, C, is indeed related to h by a third order polynomial law, i.e., equation (3.25)). Fully-coupled wave-to-wire simulations in irregular waves risk to feature sig-

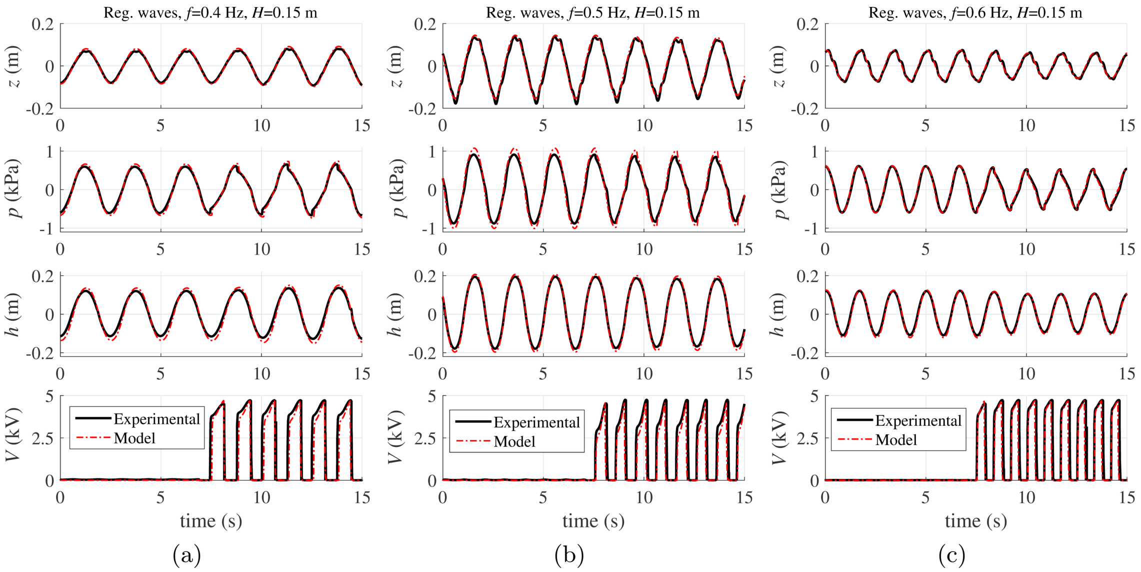

Figure S15: Comparison of experimental time-series and model predictions for z, p, h and V for three regular wave tests featuring $H = 0.15$ m and different frequencies: (a) $f = 0.4$ Hz, (b) $f = 0.5$ Hz, (c) $f = 0.6$ Hz. The CD-DEG is idle in the first half of the shown time-intervals, and actively controlled during the second half.

nificant discrepancies from the corresponding experiments owing to errors propagation (e.g., the error on the voltage profile may lead to an incorrect estimate of the electrical damping, which would thus affect the forecast of the other time-series). In contrast to this, however, here the model provides a fair agreement with the experiments, and appropriately describes the variables trends throughout the different wave trains. The most significant difference between model and experiment arises due to the presence of a threshold on the air pressure, below which the CD-DEG is not activated. Intrinsic differences between the pressure oscillation amplitudes provide that, during certain time intervals, the CD-DEG is active according to the model while it is not activated in the experiments (and vice versa). Apart from this discrepancies, the wave-to-wire model demonstrates a good robustness and reliability even in the complex scenario of electrically active operation in panchromatic waves.

In general, the model assessment in irregular waves presented so far confirms the model validity over a wide range of operating frequencies and in the presence of quickly-varying magnitude of the wave excitation.

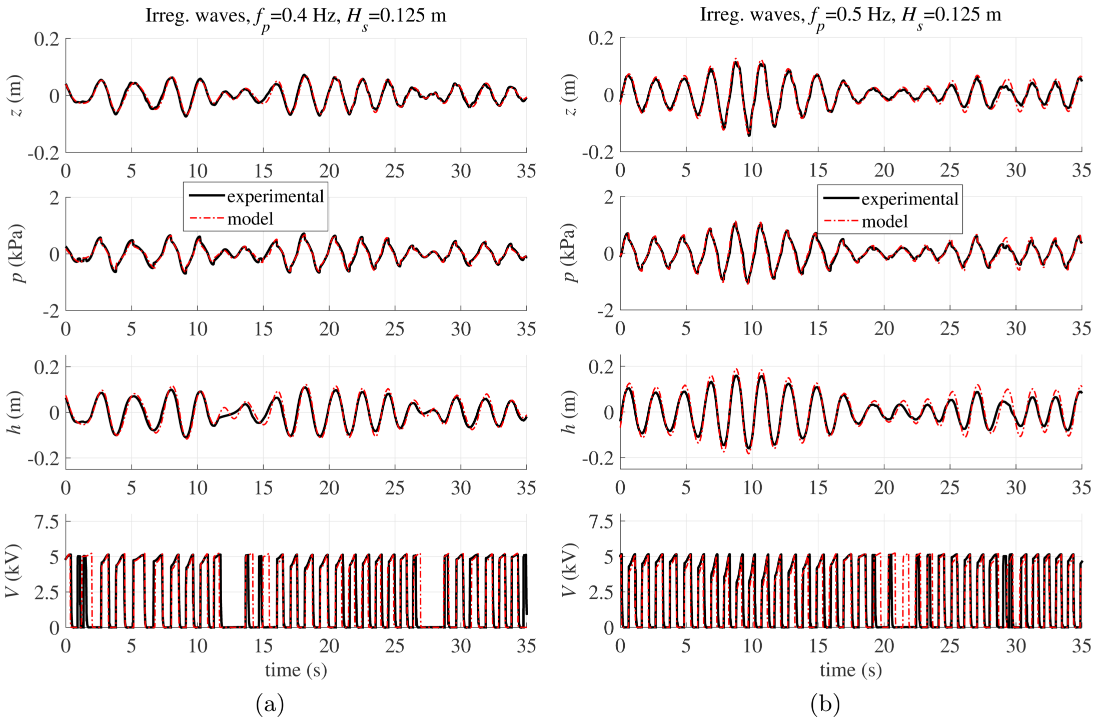

Figure S16: Comparison of relevant variables time-series (z, p, h, V) from experiments and models, for different irregular wave tests.

Extra references

[S1] Lee, C. H., and Newman, J. N.. Wamit user manual. WAMIT, Inc, 2006.

[S2] Newman, J. N. (1994). Wave effects on deformable bodies. Applied ocean research, 16(1), 47-59.

[S3] Gomes, R. P. F., Henriques, J. C. C., Gato, L. M. C., and Falcão, A. D. O. (2012). Hydrodynamic optimization of an axisymmetric floating oscillating water column for wave energy conversion. Renewable Energy, 44, 328-339.
